# Effect of rottlerin on astrocyte phenotype polarization after trimethyltin insult in the dentate gyrus of mice

**DOI:** 10.1186/s12974-022-02507-w

**Published:** 2022-06-11

**Authors:** Yeonggwang Hwang, Hyoung-Chun Kim, Eun-Joo Shin

**Affiliations:** Neuropsychopharmacology and Toxicology Program, College of Pharmacy, Kangwon National University, Chuncheon, 24341 Republic of Korea

**Keywords:** Trimethyltin, Astrocyte polarization, Rottlerin, Protein kinase Cδ, Microglia

## Abstract

**Background:**

It has been demonstrated that reactive astrocytes can be polarized into pro-inflammatory A1 phenotype or anti-inflammatory A2 phenotype under neurotoxic and neurodegenerative conditions. Microglia have been suggested to play a critical role in astrocyte phenotype polarization by releasing pro- and anti-inflammatory mediators. In this study, we examined whether trimethyltin (TMT) insult can induce astrocyte polarization in the dentate gyrus of mice, and whether protein kinase Cδ (PKCδ) plays a role in TMT-induced astrocyte phenotype polarization.

**Methods:**

Male C57BL/6 N mice received TMT (2.6 mg/kg, i.p.), and temporal changes in the mRNA expression of A1 and A2 phenotype markers were evaluated in the hippocampus. In addition, temporal and spatial changes in the protein expression of C3, S100A10, Iba-1, and p-PKCδ were examined in the dentate gyrus. Rottlerin (5 mg/kg, i.p. × 5 at 12-h intervals) was administered 3–5 days after TMT treatment, and the expression of A1 and A2 transcripts, p-PKCδ, Iba-1, C3, S100A10, and C1q was evaluated 6 days after TMT treatment.

**Results:**

TMT treatment significantly increased the mRNA expression of A1 and A2 phenotype markers, and the increased expression of A1 markers remained longer than that of A2 markers. The immunoreactivity of the representative A1 phenotype marker, C3 and A2 phenotype marker, S100A10 peaked 6 days after TMT insult in the dentate gyrus. While C3 was expressed evenly throughout the dentate gyrus, S100A10 was highly expressed in the hilus and inner molecular layer. In addition, TMT insult induced microglial p-PKCδ expression. Treatment with rottlerin, a PKCδ inhibitor, decreased Iba-1 and C3 expression, but did not affect S100A10 expression, suggesting that PKCδ inhibition attenuates microglial activation and A1 astrocyte phenotype polarization. Consistently, rottlerin significantly reduced the expression of C1q and tumor necrosis factor-α (TNFα), which has been suggested to be released by activated microglia and induce A1 astrocyte polarization.

**Conclusion:**

We demonstrated the temporal and spatial profiles of astrocyte polarization after TMT insult in the dentate gyrus of mice. Taken together, our results suggest that PKCδ plays a role in inducing A1 astrocyte polarization by promoting microglial activation and consequently increasing the expression of pro-inflammatory mediators after TMT insult.

**Supplementary Information:**

The online version contains supplementary material available at 10.1186/s12974-022-02507-w.

## Background

Astrocytes are the most abundant glial cell type in the brain, and play diverse physiological roles in maintaining brain homeostasis through regulation of ion and neurotransmitter concentrations, tissue pH, neurogenesis, synaptogenesis, and blood–brain barrier integrity [1, 2]. Astrocytes can be activated in response to brain injury or harmful stimuli, and reactive astrocytes are generally considered beneficial for neuronal survival and tissue repair by isolating damaged areas, regulating inflammatory and immune responses, maintaining glutamate and redox homeostasis, promoting tissue regeneration, and remodeling neural circuits [3–5]. In the recent decade, accumulating evidence has suggested that reactive astrocytes can be polarized into the pro-inflammatory A1 state or anti-inflammatory A2 state [6]. It has been demonstrated that pro-inflammatory cytokines (e.g., tumor necrosis factor-α (TNFα), and interferon-1α (IL-1α)) released by activated microglia stimulate A1 polarization, whereas anti-inflammatory cytokines (e.g., interleukin-10 (IL-10)) promote A2 polarization [7, 8]. Astrocyte polarization has been observed in various neurodegenerative diseases and neurotoxic conditions, such as Alzheimer’s disease (AD) [9, 10], Parkinson’s disease (PD) [9–11], ischemia [12, 13], and traumatic brain injury [14, 15]. However, little is known about the astrocyte polarization in epilepsy, although one study done by Wei et al. [16] reported the temporal and spatial profiles of A1 astrocyte polarization in the hippocampus of mice experiencing kainate-induced status epilepticus.

 Protein kinase Cδ (PKCδ) is a novel PKC isozyme [17], and has been demonstrated to play a key role in the neuroinflammation and apoptotic neuronal death in various neurodegenerative conditions [18–21]. Although the role of PKCδ in neuroinflammation after excitotoxic insult has not been well characterized, several studies, including ours, have shown that the expression of PKCδ or p-PKCδ increased in microglia, the key cells in neuroinflammatory processes, in the hippocampus following pilocarpine- or kainate-induced excitotoxicity [22–25]. Trimethyltin (TMT) is an organotin compound, and earlier studies have demonstrated that TMT exposure induces seizures and hippocampal neuronal death in humans [26–28] and rodents [29–32]. Thus, TMT has been used to establish an animal model of temporal lobe epilepsy [33, 34]. It is well known that TMT insult induces sustained convulsive behaviors and severe apoptotic neuronal death in the dentate gyrus of mice [30, 35, 36]. Our previous studies showed that PKCδ expression was induced by TMT insult in the hippocampus [37], and that PKCδ inhibition attenuated acute and delayed apoptotic cell death by promoting glutathione-related antioxidant potential and neurogenic activity [37, 38]. However, changes in astrocyte polarization or the role of PKCδ in glial activation have not been reported after in vivo TMT treatment. In the present study, we investigated the temporal and spatial changes in astroglial activation and polarization after TMT insult in the dentate gyrus of mice. In addition, we investigated the effect and mechanisms of the PKCδ inhibitor rottlerin on astrocyte polarization following TMT exposure.

## Methods

### Animals and treatments

All animals were treated in accordance with the National Institutes of Health (NIH) Public Health Service Policy on Humane Care and Use of Laboratory Animals (2015 Edition; grants.nih.gov/grants/olaw/references/PHSPolicyLabAnimals.pdf) and in accordance with the Institute for Laboratory Animal Research (ILAR) Guidelines for the Care and Use of Laboratory Animals (8th Edition; grants.nih.gov/grants/olaw/Guide-for-the-care-and-use-of-laboratory-animals.pdf). The animal experimental procedure was approved by the Institutional Animal Care and Use Committee (IACUC) of Kangwon National University (#KW-180706-2 and #KW-210817-1). Eight-week-old male C57BL/6N mice (Orient Bio, Inc., Charles River Technology, Seoul, Republic of Korea) were maintained on a 12/12 h light/dark cycle and fed ad libitum*.* They were adapted to these conditions for 2 weeks prior to the experiment. In the present study, male mice were used to avoid the influence of the estrus cycle of female mice, because previous studies have suggested that estrogen or the estrus cycle could affect astroglial and microglial activation, and consequently neuroinflammation [39–42].

TMT (Sigma-Aldrich, St. Louis, MO, U.S.A.) was dissolved in sterile saline. Rottlerin (Tocris Bioscience, Bristol, U.K.) was dissolved in dimethyl sulfoxide (DMSO), and then diluted in sterile saline. The final concentration of DMSO was 10% (v/v). All reagents were prepared immediately before use.

In the first experiment, temporal and spatial changes in astrocyte polarization were evaluated after TMT treatment in the dentate gyrus. Mice were administered TMT (2.6 mg/kg, i.p.) and euthanized with cervical dislocation for real-time reverse transcription-polymerase chain reaction (RT-PCR) analysis or were perfused under urethane (1.5 g/kg, i.p.) anesthesia for histological evaluation 1, 2, 6, 10, and 14 days after TMT administration. The number of mice in each group is summarized in Table 1.

**Table 1:** Number of mice in each group

	Group	Number of mice for histological evaluation	Number of mice for RT-PCR and Western blot analyses
Experiment I	Saline	4	6
	1 d	4	6
	2 d	4	5
	6 d	4	6
	10 d	4	6
	14 d	-	6
Experiment II	Vehicle + Saline	4	6^#^
	Rottlerin + Saline	4	6^#^
	Vehicle + TMT	5	6^#^
	Rottlerin + TMT	5	6^#^
Total number of mice		97	

^#^One hemisphere was used for RT-PCR and the other hemisphere was used for Western blot analysis

In the second experiment, the effect of rottlerin on TMT-induced astrocyte polarization was examined in the dentate gyrus. Since changes in the protein expression of A1 and A2 astrocyte markers were most pronounced 6 days after TMT insult in the first experiment, and to avoid the direct effect of rottlerin on convulsive behaviors and acute neurotoxicity, rottlerin treatment was performed near and after the end of convulsion (late- and post-ictal treatment). Mice received a single injection of TMT (2.6 mg/kg, i.p.), and convulsive behaviors were measured 2 days after TMT injection. Mice were then grouped based on convulsive behaviors (each group had equivalent average convulsive behaviors), and treatment with rottlerin (5 mg/kg, i.p. × 5 at 12-h intervals) or vehicle (10% DMSO diluted in sterile saline) was initiated 3 days after TMT administration. Our previous study showed that TMT-induced convulsive behaviors peaked 2 days after TMT treatment, and then markedly decreased 3 days after TMT injection, and returned to near control levels 4 days after TMT administration [38]. Twenty-four hours after the final treatment with rottlerin (6 days after TMT administration), mice were euthanized with cervical dislocation for Western blot and RT-PCR analyses or were perfused under urethane (1.5 g/kg, i.p.) anesthesia for histological evaluation. The number of mice in each group is summarized in Table 1. The dose of rottlerin was determined based on previous studies [38, 43]. The experimental design is shown in Fig. 5A.

### Measurement of convulsive behaviors

Convulsive impulse counts were measured for 3 min, as described in our previous study [37, 38], using a convulsion meter (CONVULS-1; Columbus Instruments, Columbus, OH, USA) that comprises an acrylic box and a detection sensor.

### Real-time RT-PCR

Real-time RT-PCR was performed as described previously [44]. Total RNA was isolated from the hippocampus using the RNeasy Mini kit (Qiagen, Valencia, CA, USA). Extracted RNA (1 μg) was reverse transcribed into cDNA by iScript™ Advanced cDNA Synthesis Kit (Bio-Rad Laboratories, Inc., Hercules, CA, USA). Equal amount of cDNA was added to PCR reaction mixture, containing 10 pmol of each primer and SsoAdvanced™ Universal SYBR Green Supermix (Bio-Rad Laboratories, Inc.). They were amplified with CFX96 Touch real time PCR system (Bio-Rad Laboratories, Inc.). The reference gene (GAPDH) and target gene from each sample were run in parallel in the same plate with the same amount of cDNA. Real-time cycling parameters were as follows: activation of DNA polymerase at 95 °C for 3 min, 40 cycles of denaturation at 95 °C for 10 s, and annealing and extension for 30 s. Primer sequences and annealing temperatures are listed in Additional file 1: Table S1 (see Additional file 1). The relative mRNA expression level was quantified using the 2^−ΔΔCt^ method [45].

### Immunohistochemistry

Mice were anesthetized with urethane (1.5 g/kg, i.p.), and perfused transcardially with 50 mL of phosphate buffered saline (PBS; pH 7.4), followed by 30 mL of 4% paraformaldehyde (Sigma-Aldrich) in PBS at a rate of 10 ml/min. The brains were post-fixed with the same fixative solution for 24 h at 4 °C, and cryoprotected with 30% sucrose in PBS at 4 °C until sunk. The brains were then sectioned using a horizontal sliding microtome into 35 μm transverse free floating sections. Immunohistochemistry was performed as described in our previous study [25, 38]. Hippocampal sections were blocked and permeabilized by incubation with PBS containing 0.3% hydrogen peroxide (Sigma-Aldrich), and then PBS containing 0.25% bovine serum albumin (BSA, Sigma-Aldrich), 4% normal serum (Vector Laboratories, Burlingame, CA, U.S.A.) and 0.4% Triton X-100. After incubation with primary antibody against glial fibrillary acidic protein (GFAP, 1:500; #MAB3402, Millipore, Temecula, CA, USA), complement component 3 (C3, 1:100; #ab11862, Abcam, Cambridge, MA, USA), S100A10 (1:200; #AF2377, R&D Systems, Minneapolis, MN, USA), Iba-1 (1:500, #019-19741; Wako Pure Chemical Industries, Chuo-ku, Osaka, Japan), p-PKCδ (1:500; #sc-365969, Santa Cruz Biotechnology, Inc., Santa Cruz, CA, USA), or complement component 1q (C1q, 1:250; #ab11861, Abcam) for 48 h at 4 °C, sections were incubated with biotinylated secondary antibody (1:1,000; Vector Laboratories) and then avidin–biotin-peroxidase complex (Vector Laboratories) for 1 h at room temperature. Sections were rinsed with PBS containing 0.25% BSA for 10 min twice between each step. DAB was used as a chromogen. Digital images were obtained using a microscope (BX51, Olympus) and a digital microscope camera (DP72, Olympus) at 50 × , 100 × , or 200 × magnification. ImageJ version 1.53v software with Fiji plug-in packages (National Institutes of Health, USA) was employed to analyze immunoreactivity in the dentate gyrus.

The morphology of Iba-1-positive cells was analyzed by measuring cell body size measurement and cell skeleton analysis, according to previous studies [46, 47], using ImageJ version 1.53v software with Fiji plug-in packages (National Institutes of Health). Briefly, digital images of Iba-1-immunostained sections were obtained at × 400 magnification. Each section was acquired in four focal 4-planes, and these images were stacked and integrated into one image. All pixels that were darker than the background were selected by threshold command to select the whole cell area. Cell bodies were manually selected by threshold adjustment and wand tool of ImageJ (Additional file 2: Fig. S3A, see Additional file 2). The “Analyze Particles” command was performed to measure the cell size and cell body size. The number of cell bodies was counted to normalize the cell size and cell body size per cell. For skeleton analysis, the images were binarized, skeletonized, and then analyzed using the “Analyze Skeleton” command (Additional file 2: Fig. S3B, see Additional file 2). The number of branches, number of junctions, average branch length, and summed branch length were determined.

### Immunofluorescence and confocal microscopy

Double-labeling immunofluorescence was carried out as described in our previous study [38]. Hippocampal sections were blocked and permeabilized by incubation with PBS containing 0.25% BSA (Sigma-Aldrich), 4% normal serum (Vector Laboratories) and 0.4% Triton X-100 for 30 min at room temperature, and then incubated in a mixture of GFAP (1:500; #MAB3402, Millipore) and C3 (1:100; #ab11862, Abcam) or S100A10 (1:200; #AF2377, R&D Systems), a mixture of C3 (1:100; #ab11862, Abcam) and S100A10 (1:200; #AF2377, R&D Systems), or a mixture of Iba-1 (1:500, #019-19741; Wako Pure Chemical Industries) and p-PKCδ (1:500; #sc-365969, Santa Cruz Biotechnology, Inc.) for 48 h at 4 °C. After incubation with a mixture of Alexa Fluor 488- and Alexa Fluor 594-conjugated secondary antibodies (1:200; Invitrogen, Carlsbad, CA, U.S.A.), sections were counterstained with 4′,6-diamidino-2-phenylindole (DAPI, Sigma-Aldrich) for 30 min at room temperature. Sections were rinsed with PBS for 10 min twice between each step. Digital images were acquired at 200 × magnification, using a confocal laser scanning microscope (LSM 880 with Airyscan; Carl Zeiss AG, Oberkochen, Germany; The Central Laboratory, Chuncheon Campus, Kangwon National University, Republic of Korea). ImageJ version 1.53v software with Fiji plug-in packages (National Institutes of Health, USA) was employed to analyze the co-localization of C3 and S100A10 in the dentate gyrus 6 days after TMT treatment. Specifically, the Pearson’s correlation coefficients, thresholded Mander’s co-localization coefficient (tM), and Costes P-value were recorded using “Coloc 2” plug-in (https://imagej.net/plugins/coloc-2).

### Western blot analysis

Western blot analysis was performed as described in our previous study [25, 38]. Hippocampal tissues were homogenized in lysis buffer containing 200 mM Tris HCl (pH 6.8), 1% SDS, 5 mM EGTA, 5 mM ethylenediaminetetraacetic acid (EDTA), 10% glycerol, 1 × phosphatase inhibitor cocktail I (Sigma-Aldrich), and 1 × protease inhibitor cocktail (Sigma-Aldrich). The lysates were centrifuged for 20 min at 13,000 × *g* at 4 °C, and supernatant was used for Western blotting. Protein concentration was determined using the BCA Protein Assay kit (Thermo Scientific, Rockford, IL, U.S.A.). Proteins (30–50 µg/lane) were separated by 10% sodium dodecyl sulfate (SDS)-polyacrylamide gel electrophoresis (PAGE) and transferred onto the PVDF membranes. The membranes were blocked by incubation with 5% non-fat milk for 30 min, and then incubated overnight at 4 °C with primary antibody against interleukin-1β (IL-1β, 1:1,000; #MAB401, R&D Systems), TNFα (1:500; # AF-410-NA, R&D Systems), or β-actin (1:300,000; #A5441, Sigma-Aldrich). Membranes were then incubated with horseradish peroxidase-conjugated secondary anti-goat IgG (1:1,000; Sigma-Aldrich) for 1 h at room temperature. Subsequent visualization was achieved using an enhanced chemiluminescence system (ECL plus®, GE Healthcare, Piscataway, NJ, USA). Relative intensities of the bands were analyzed by PhotoCapt MW (version 10.01 for Windows; Vilber Lourmat, Marne la Vallée, France), and then normalized to the intensity of β-actin.

### Terminal deoxynucleotidyl transferase‑mediated dUTP nick end‑labeling (TUNEL) staining

TUNEL staining was conducted using the In situ Cell Death Detection Kit, POD (Roche, Mannheim, Germany) according to the manufacturer's protocol. Briefly, hippocampal sections were permeabilized with 0.1% Triton X-100 in 0.1% sodium citrate. After incubation with TUNEL reaction mixture at 37 °C for 60 min, sections were incubated with converter-POD solution at 37 °C for 30 min in the humidifier chamber. 3,3’-diaminobenzidine (DAB; Sigma-Aldrich) was used as a chromogen. Counterstaining was done with 0.5% cresyl violet dye (Sigma-Aldrich) for 2 min. Digital images were acquired at 100 × and 200 × magnification using a microscope (BX51; Olympus) and a digital microscope camera (DP72; Olympus), as described in our previous study [37, 38]. TUNEL-positive cells in the dentate gyrus were analyzed by ImageJ version 1.53v software with Fiji plug-in packages (National Institutes of Health, USA).

### Statistical analyses

Data were analyzed using IBM SPSS ver. 24.0 (IBM Corporation, Armonk, NY, USA). Unpaired *t*-test, one-way (time points) or two-way ANOVA (TMT × rottlerin) was employed for the statistical analyses. Post hoc Fisher's least significant difference (LSD) pairwise comparisons were then conducted. *P*-value < 0.05 was deemed to be significant. The results of ANOVA were summarized in Additional file 1: Tables S2–S5 (see Additional file 1).

## Results

### TMT treatment induced astroglial activation and phenotypic changes in the dentate gyrus of mice

We first examined the temporal and spatial patterns of astroglial activation and phenotypic changes after TMT treatment in the dentate gyrus of mice. As shown in Additional file 2: Fig. S1 (see Additional file 2), the expression of GFAP, an astrocyte marker, increased at 2 days, and this increase was much more pronounced at 6 and 10 days after TMT treatment. In quantitative analysis, one-way ANOVA found a significant effect of TMT on the GFAP-immunoreactivity, in terms of integrated density and area (Additional file 1: Table S5, see Additional file 1). Post hoc test showed that GFAP expression significantly increased at 2 days after TMT treatment (area: *P* < 0.01 vs. saline). This increase in GFAP expression was more pronounced at 6 days (integrated density and area: *P* < 0.01 vs. saline), and was maintained at least until 10 days after TMT treatment (integrated density and area: *P* < 0.01 vs. saline), suggesting that TMT treatment resulted in marked astroglial activation in the dentate gyrus of mice (Additional file 2: Fig. S1, see Additional file 2).

**Fig. 1:**
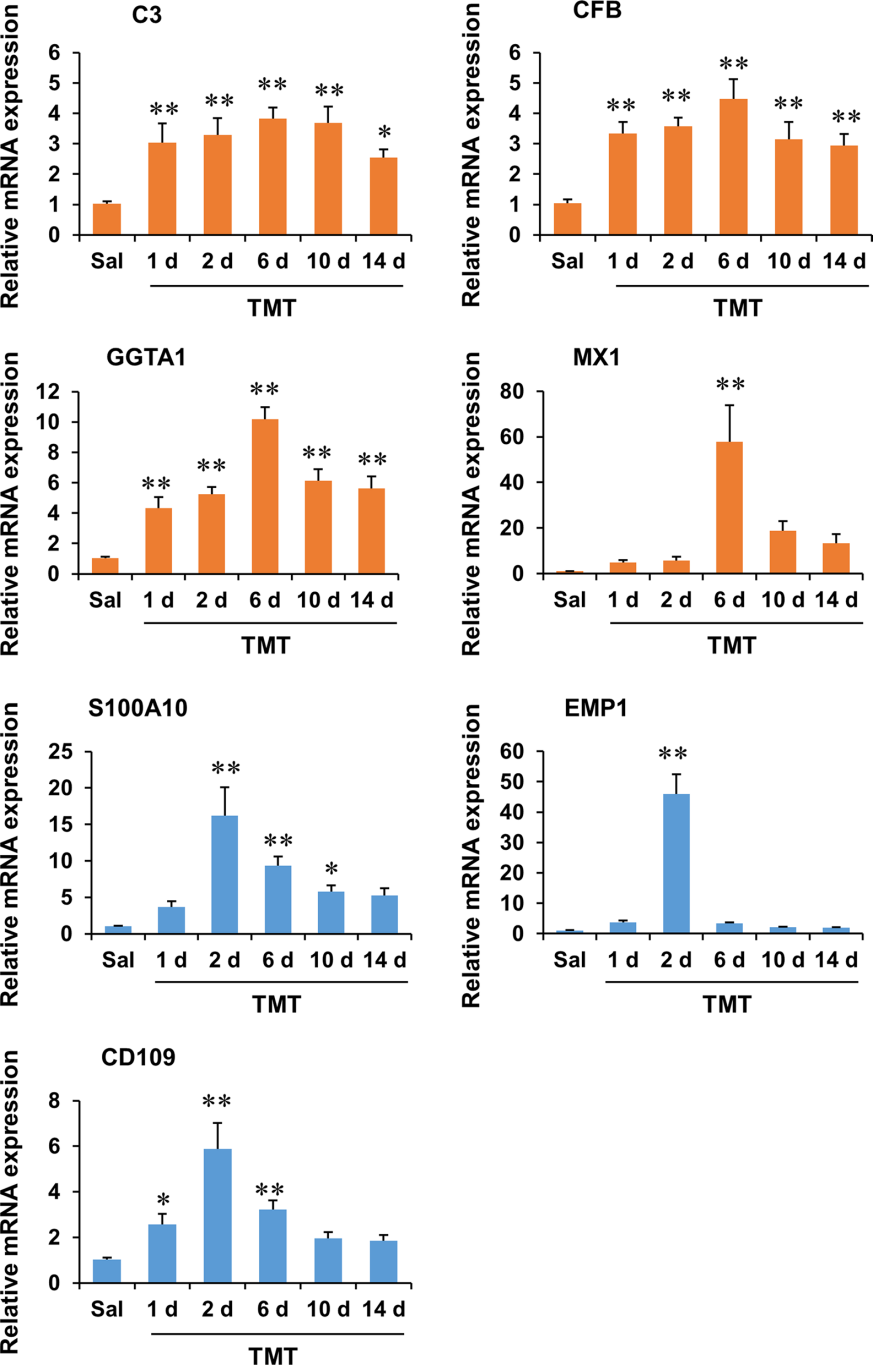
Temporal changes in the mRNA expression of A1 and A2 astrocyte phenotype markers after TMT treatment in the dentate gyrus of mice. C3, CFB, GGTA1, and MX1 are A1 phenotype markers. S100A10, EMP1, and CD109 are A2 phenotype markers. Sal, Saline. Each value is the mean ± S.E.M. of 5 (Saline, 1 d, 6 d, 10 d, and 14 d) or 6 (2 d) mice. ^*^P < 0.05, ^**^P < 0.01 vs. Saline (one-way ANOVA followed by Fisher’s LSD pairwise comparisons test)

Temporal changes in the mRNA expression of astrocyte phenotype markers were evaluated by real-time RT-PCR in the hippocampus. One-way ANOVA revealed a significant effect of TMT on the mRNA expression of A1 phenotype markers (C3, complement factor B (CFB), glycoprotein galactosyltransferase α1, 3 (GGTA1), and MX1) and A2 phenotype markers (S100A10, epithelial membrane protein 1 (EMP1), and CD109) (Additional file 1: Table S1, see Additional file 1). Post hoc test showed that the mRNA expression of A1 phenotype markers C3, CFB, and GGTA1 significantly increased 1 day after TMT treatment. The expression levels of these genes peaked at 6 days and remained increased until 14 days after TMT treatment. MX1 mRNA expression also peaked at 6 days after TMT treatment. The mRNA expression of A2 phenotype markers significantly increased and peaked at 2 days, and gradually decreased to near control levels until 14 days after TMT treatment (Fig. 1).

**Fig. 2:**
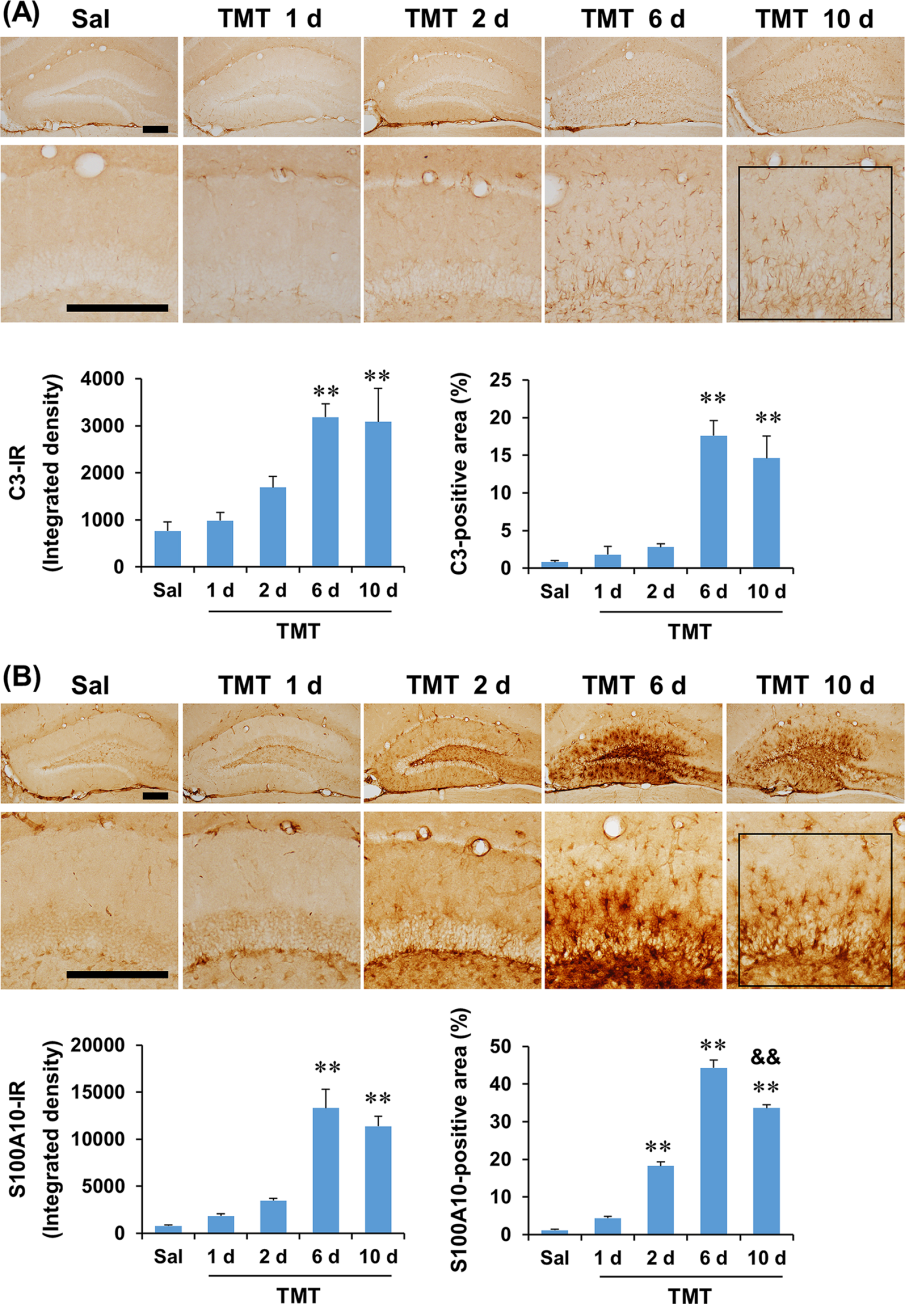
Temporal and spatial changes in C3 and S100A10 expression after TMT treatment in the dentate gyrus of mice. **A** C3 expression. **B** S100A10 expression. Square boxes in **A** and **B** indicate the region of interest for quantification. Sal, Saline. Each value is the mean ± S.E.M. of 4 (Saline, 1 d, 2 d, 6 d, 10 d, and 14 d) mice. ^*^P < 0.05, ^**^P < 0.01 vs. Saline; ^&&^*P* < 0.01 vs. TMT 6 d (one-way ANOVA followed by Fisher’s LSD pairwise comparisons test). Scale bar = 200 µm

**Fig. 3:**
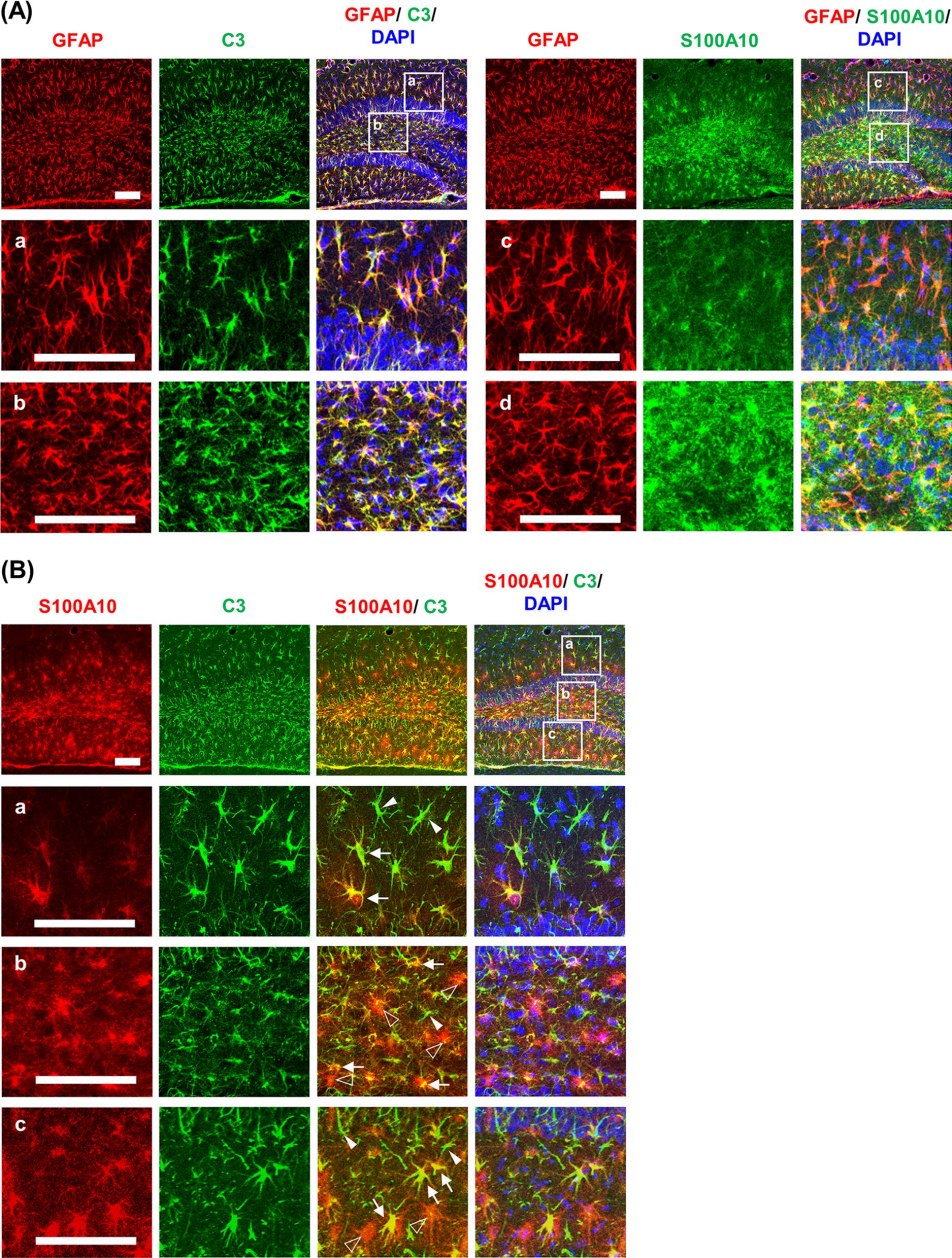
Double-labeling immunofluorescence of C3 and S100A10 6 days after TMT treatment. **A** Co-localization of GFAP and C3 or S100A10 6 days after TMT treatment. **B** Co-localization of C3 and S100A10 6 days after TMT treatment. Pearson’s correlation coefficient = 0.413 ± 0.0377; Thresholded Mander’s co-localization coefficient (tM) for C3 = 0.523 ± 0.0190; tM for S100A10 = 0.423 ± 0.0123; Costes P-value = 1.000 ± 0.000 (*n* = 4). Arrows indicate astrocytes that are positive for both C3 and S100A10. Closed arrowheads indicate astrocytes that are positive for C3. Open arrowheads indicate astrocytes that are positive for S100A10. Scale bar = 100 µm

Temporal and spatial expression patterns of the representative A1 phenotype marker C3 and A2 phenotype marker S100A10 after TMT treatment in the dentate gyrus were evaluated by immunohistochemistry (Fig. 2). TMT treatment appeared to markedly increase C3 and S100A10 expression at 6 and 10 days after the injection. In quantitative analysis, one-way ANOVA found a significant effect of TMT on C3- and S100A10-immunoreactivity, in terms of integrated density and area (Additional file 1: Table S2, see Additional file 1). Post hoc test showed that C3 expression significantly increased at 6 and 10 days after TMT treatment (integrated density and area: *P* < 0.01 vs. saline) (Fig. 2A). S100A10 expression significantly increased at 2 days (area: *P* < 0.01 vs. saline), and peaked at 6 days after TMT treatment (integrated density and area: *P* < 0.01 vs. saline). TMT-induced S100A10 expression then significantly decreased at 10 days compared to 6 days after treatment (area: TMT 10 d vs. TMT 6 d, *P* < 0.01), suggesting that A1 astrocyte polarization is more persistent than A2 astrocyte polarization after TMT insult (Fig. 2B). Since C3 and S100A10 expression was most evident at 6 days, double-labeling immunofluorescence was performed at this time point to characterize the spatial expression pattern of C3 and S100A10 induced by TMT. C3-positive immunofluorescence was evenly distributed throughout the dentate gyrus, and most GFAP-positive astrocytes co-localized with C3. On the other hand, S100A10-positive immunofluorescence was more intense in GFAP-positive astrocytes located in the hilus, granular layer, and inner molecular layer, but less intense in GFAP-positive astrocytes in the outer molecular layer (Fig. 3A). A brief co-localization analysis of the representative photomicrographs in Fig. 3A showed that 85.4% of GFAP-positive cells were co-localized with C3 (tM for GFAP = 0.854, tM for C3 = 0.862) and 57.0% of GFAP-positive cells were co-localized with S100A10 (tM for GFAP = 0.570, tM for S100A10 = 0.500). C3 and S100A10 have been suggested as specific and exclusive markers of A1 and A2 phenotypes, respectively [48, 49]. However, the sum of the proportions of C3-positive and S100A10-positive cells was greater than 100% of GFAP-positive astrocytes in the present study, suggesting that astrocytes could express C3 and S100A10 simultaneously. Thus, we evaluated whether C3-positive astrocytes were co-localized with S100A10-positive astrocytes in the dentate gyrus at 6 days after TMT treatment. Interestingly, a considerable proportion of astrocytes expressed both C3 and S100A10. In the co-localization analysis, 52.3% of C3-positive cells were co-localized with S100A10 [tM for C3 = 0.523 ± 0.0190 (*n* = 4)] and 42.3% of S100A10-positive cells were co-localized with C3 [tM for S100A10 = 0.423 ± 0.0123 (*n* = 4)]. The findings indicate significant co-localization of C3 and S100A10 expression induced by TMT treatment in the dentate gyrus of mice (Fig. 3B).

### Temporal pattern of microglial activation was parallel with that of astroglial activation and phenotypic changes after TMT treatment in the dentate gyrus of mice

**Fig. 4:**
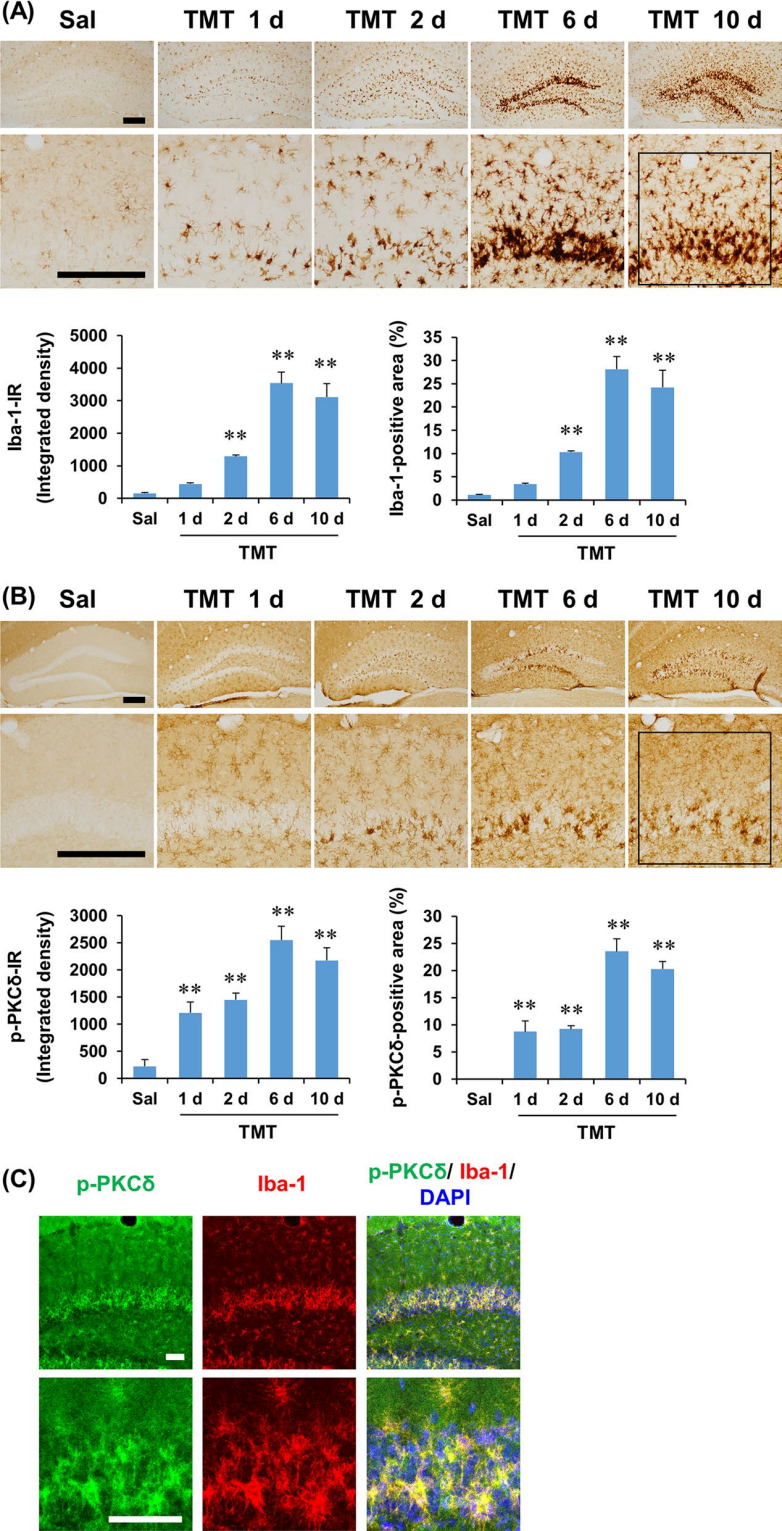
Temporal and spatial changes in Iba-1 and p-PKCδ expression after TMT treatment in the dentate gyrus of mice. **A** Iba-1 expression. **B** p-PKCδ expression. **C** Co-localization of Iba-1 and p-PKCδ 6 days after TMT treatment. Square boxes in A and B indicate the region of interest for quantification. Sal, Saline. Each value is the mean ± S.E.M. of 4 (Saline, 1 d, 2 d, 6 d, 10 d, and 14 d) mice. ^**^P < 0.01 vs. Saline (one-way ANOVA followed by Fisher’s LSD pairwise comparisons test). Scale bar = 200 (A and B) or 50 (C) µm

Activated microglia have been suggested to play an important role in astrocyte phenotype polarization [7, 8]. Therefore, we next examined the temporal pattern of Iba-1 expression in the dentate gyrus after TMT treatment. As shown in Fig. 4A, a small number of activated microglia was observed mainly in the granular cell layer at 1 day after TMT treatment. These changes became more obvious at 2 days and were markedly pronounced at 6 and 10 days after TMT insult. In quantitative analysis, ANOVA found a significant effect of TMT on Iba-1 expression, in terms of integrated density and area (Additional file 1: Table S2, see Additional file 1). Post hoc test indicated that Iba-1 expression significantly increased at 2 days (integrated density and area: *P* < 0.01 vs. saline), and this increase in Iba-1 expression was more pronounced at 6 and 10 days after TMT (integrated density and area: *P* < 0.01 vs. saline), suggesting that the temporal pattern of microglial activation is consistent with that of astroglial activation and phenotypic alteration, particularly A1 phenotype polarization, in the dentate gyrus of mice after TMT treatment (Fig. 4A).

### TMT treatment induced microglial p-PKCδ expression in the dentate gyrus of mice

PKCδ has been demonstrated to be involved in microglial activation under various excitotoxic conditions [22–25]. Since little is known about the role of PKCδ in microglial activation and neuroinflammatory changes after TMT insult, we examined the temporal changes in p-PKCδ expression after TMT treatment in the dentate gyrus of mice. The basal expression pattern of p-PKCδ in the hippocampus is shown in Additional file 2: Fig. S2 (see Additional file 2); p-PKCδ expression was intense in the CA1 subfield and moderate in the CA2 and CA3 subfields, but negligible in the dentate gyrus. As shown in Fig. 4B, p-PKCδ expression was induced 1 and 2 days after TMT treatment in the dentate gyrus. TMT-induced p-PKCδ expression was more pronounced in the granular cell layer and molecular layer of the dentate gyrus at 6 and 10 days after treatment. The majority of p-PKCδ-positive cells appeared to be microglia in the dentate gyrus of TMT-treated mice. In quantitative analysis, ANOVA revealed a significant effect of TMT on p-PKCδ expression, in terms of integrated density and area (Additional file 1: Table S2, see Additional file 1). Post hoc test indicated that p-PKCδ expression significantly increased at 1 and 2 days (integrated density and area: *P* < 0.01 vs. saline), and became more evident at 6 and 10 days after TMT treatment (integrated density and area: *P* < 0.01 vs. saline) (Fig. 4B). Double-labeling immunofluorescence indicated that p-PKCδ-positive immunofluorescence was mainly localized in Iba-1-positive cells at 6 days after TMT insult, suggesting that TMT treatment induces microglial p-PKCδ expression (Fig. 4C).

### PKCδ inhibitor rottlerin attenuated microglial activation induced by TMT in the dentate gyrus of mice

**Fig. 5:**
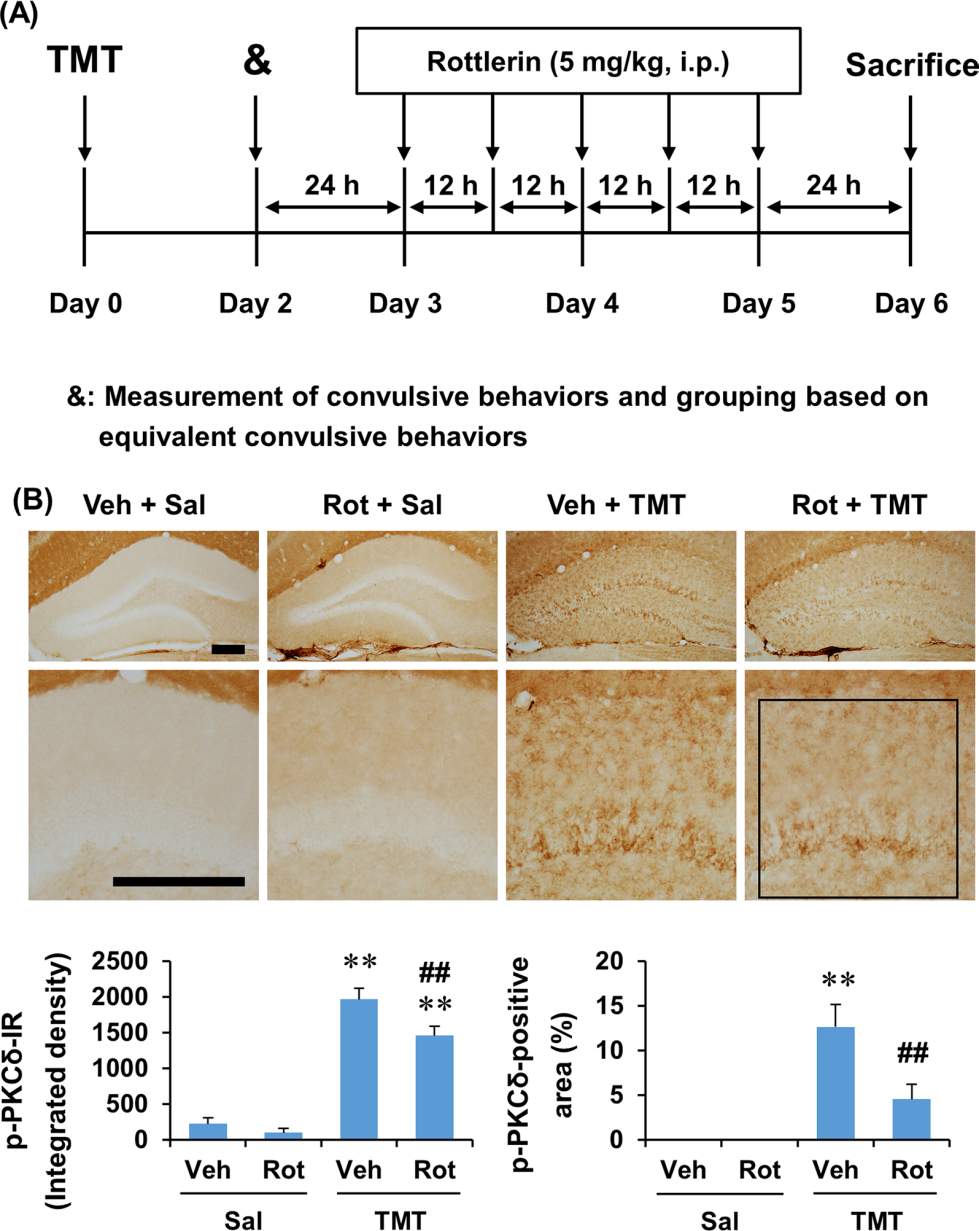
Experimental schedule and the effect of rottlerin on the expression of p-PKCδ in the dentate gyrus of mice 6 days after TMT treatment. **A** Experimental schedule to investigate the effect of rottlerin on astroglial activation and phenotypic changes induced by TMT. Rottlerin (5.0 mg/kg, i.p. × 5 at 12-h intervals) administration was began 3 days after TMT treatment, and mice were euthanized with cervical dislocation for Western blot and RT-PCR analyses or were perfused under urethane anesthesia for histological evaluation 1 day after the final administration of rottlerin. **B** Effect of rottlerin on p-PKCδ expression. Square box in B indicates the region of interest for quantification. Veh, Vehicle. Sal, Saline. Rot, Rottlerin. Each value is the mean ± S.E.M. of 4 (Vehicle + Saline and Rottlerin + Saline) or 5 (Vehicle + TMT and Rottlerin + TMT) mice. ^*^P < 0.05, ^**^P < 0.01 vs. corresponding Saline; ^##^*P* < 0.01 vs. Vehicle + Saline (two-way ANOVA followed by Fisher’s LSD pairwise comparisons test). Scale bar = 200 µm

Since TMT treatment induced microglial p-PKCδ expression, we next examined the effect of late- and post-ictal treatment with rottlerin on Iba-1 expression at 6 days after TMT treatment (Fig. 5A). Two-way ANOVA found a significant effect of TMT and rottlerin and a significant rottlerin × TMT interaction on p-PKCδ and Iba-1 expression, in terms of integrated density and area (Additional file 1: Table S2, see Additional file 1). Post hoc test indicated that rottlerin significantly attenuated TMT-induced p-PKCδ expression at 6 days (integrated density and area: *P* < 0.01 vs. vehicle + TMT), and rottlerin-mediated attenuation of p-PKCδ expression appeared to be more pronounced in the molecular layer than in the granular cell layer of dentate gyrus treated with TMT (Fig. 5B).

**Fig. 6:**
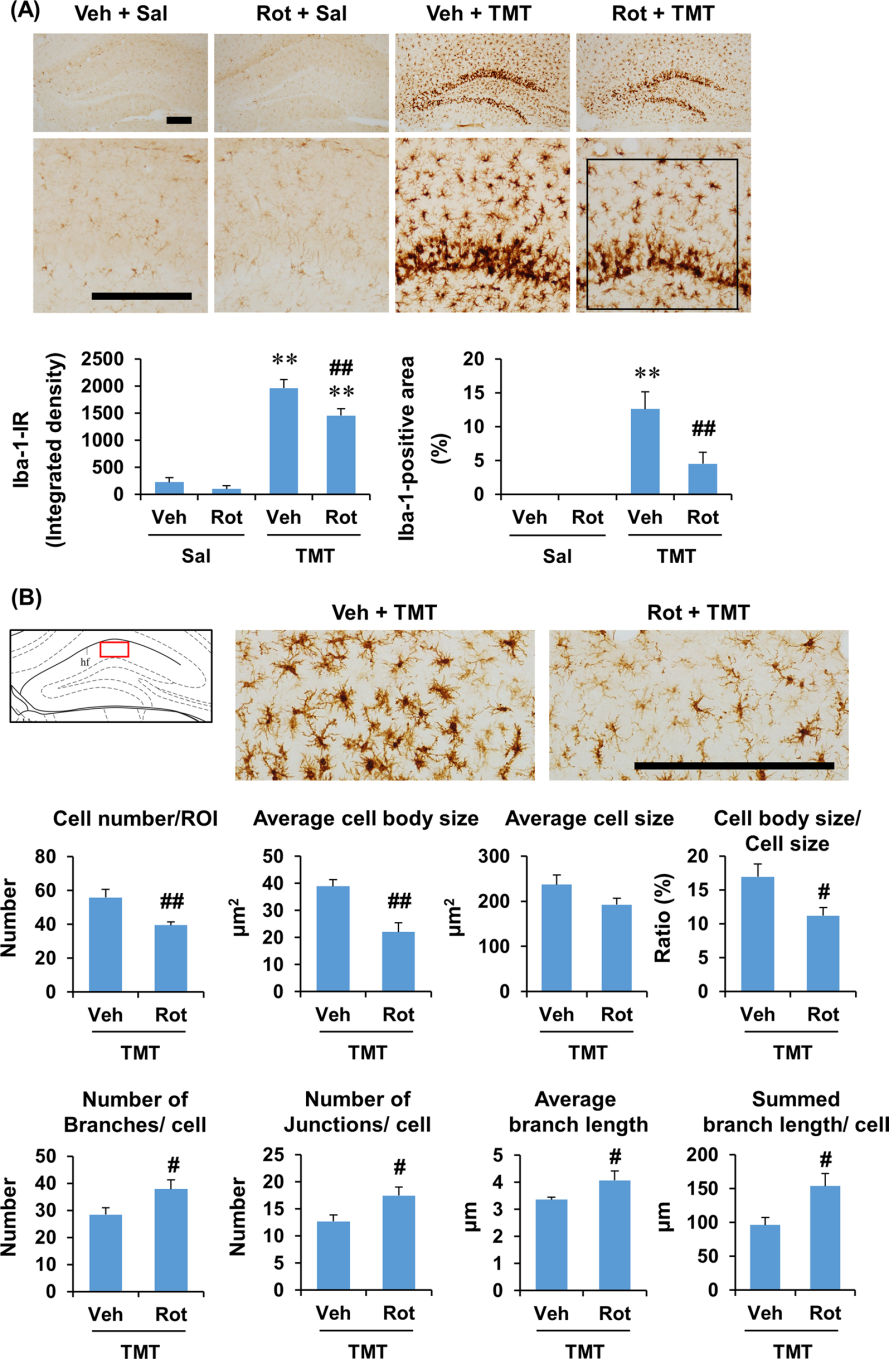
Experimental schedule and the effect of rottlerin on the expression of Iba-1 in the dentate gyrus of mice 6 days after TMT treatment. **A** Effect of rottlerin on Iba-1 immunoreactivity. **B** Effect of rottlerin on the morphology of Iba-1-positive cells in the dentate gyrus of TMT-treated mice. Square boxes in A and B indicate the region of interest (ROI) for quantification. Veh, Vehicle. Sal, Saline. Rot, Rottlerin. Each value is the mean ± S.E.M. of 4 (Vehicle + Saline and Rottlerin + Saline) or 5 (Vehicle + TMT and Rottlerin + TMT) mice. ^*^P < 0.05, ^**^P < 0.01 vs. corresponding Saline; ^##^*P* < 0.01 vs. Vehicle + Saline [two-way ANOVA followed by Fisher’s LSD pairwise comparisons test **A** or unpaired *t*-test (**B**)]. Scale bar = 200 µm

Consistently, rottlerin significantly attenuated Iba-1 expression at 6 days after TMT treatment (integrated density and area: *P* < 0.01 vs. vehicle + TMT), suggesting that PKCδ inhibition attenuates TMT-induced Iba-1 expression in the dentate gyrus. Similar to p-PKCδ expression, the effect of rottlerin appeared to be more prominent in the molecular layer than in the granular cell layer in TMT-treated mice (Fig. 6A). In addition, the number of Iba-1-positive microglia was significantly decreased (*t*_8_ = 3.110, *P* < 0.01 vs. vehicle + TMT) by rottlerin in the dentate gyrus of TMT-treated mice, suggesting that rottlerin attenuates microglial proliferation or migration in response to TMT insult (Fig. 6B). We further analyzed the effect of rottlerin on microglial morphology after TMT treatment. In cell morphology analysis of Iba-1-positive microglia, rottlerin significantly decreased cell body size (*t*_8_ = 3.996, *P* < 0.01 vs. vehicle + TMT) and cell body size/cell size ratio (*t*_8_ = 2.617, *P* < 0.05 vs. vehicle + TMT). Significant increases were observed in the number of branches (*t*_8_ = 2.222, *P* < 0.05 vs. vehicle + TMT), number of junctions (*t*_8_ = 2.364, *P* < 0.05 vs. vehicle + TMT), average branch length (*t*_8_ = 2.023, *P* < 0.05 vs. vehicle + TMT), and summed branch length (*t*_8_ = 2.655, *P* < 0.01 vs. vehicle + TMT) after rottlerin administration in the dentate gyrus of TMT-treated mice (Fig. 6B). It has been demonstrated that the cell body size increases and the number and length of branches decrease as microglia are activated [50, 51]. Thus, these results suggest that rottlerin attenuates microglial activation as well as microglial proliferation or migration in the dentate gyrus of TMT-treated mice (Fig. 6).

### PKCδ inhibitor rottlerin attenuated A1 astrocyte polarization induced by TMT in the dentate gyrus of mice

We then examined the effect of rottlerin on astroglial activation and their phenotypic polarization after TMT insult. As shown in Additional file 2: Fig. S5 (see Additional file 2), rottlerin did not appear to affect GFAP expression at 6 days after TMT treatment. In quantitative analysis, two-way ANOVA revealed a significant effect of TMT, but not of rottlerin, on GFAP expression (Additional file 1: Table S5, see Additional file 1).

**Fig. 7:**
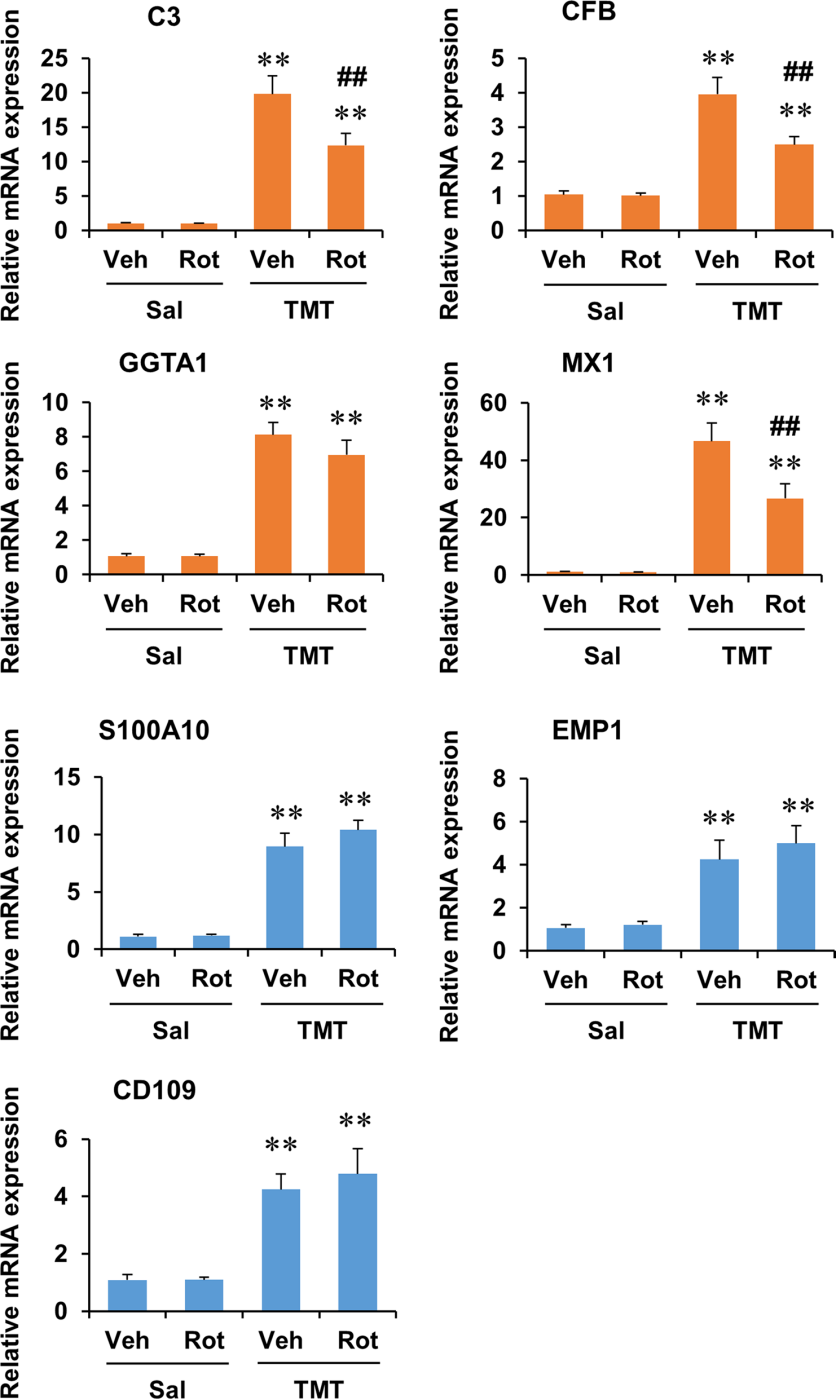
Effect of rottlerin on the mRNA expression of astrocyte phenotype markers in the dentate gyrus of mice 6 days after TMT treatment. C3, CFB, GGTA1, and MX1 are A1 phenotype markers. S100A10, EMP1, and CD109 are A2 phenotype markers. Veh, Vehicle. Sal, Saline. Rot, Rottlerin. Each value is the mean ± S.E.M. of 6 (Vehicle + Saline, Rottlerin + Saline, Vehicle + TMT, and Rottlerin + TMT) mice. ^**^P < 0.01 vs. corresponding Saline; ^##^*P* < 0.01 vs. Vehicle + Saline (two-way ANOVA followed by Fisher’s LSD pairwise comparisons test)

In real-time RT-PCR analysis of the mRNA expression of astrocyte phenotype markers, two-way ANOVA indicated the significant effect of TMT and rottlerin and a significant TMT × rottlerin interaction on the mRNA expression of A1 phenotype markers, including C3, CFB, and MX1 (Additional file 1: Table S3, see Additional file 1). However, rottlerin did not produced a significant effect on the mRNA expression of A2 phenotype markers. Post hoc test indicated that rottlerin significantly decreased the mRNA expression of C3, CFB, and MX1 at 6 days after TMT treatment (*P* < 0.01 vs. vehicle + TMT) (Fig. 7).

**Fig. 8:**
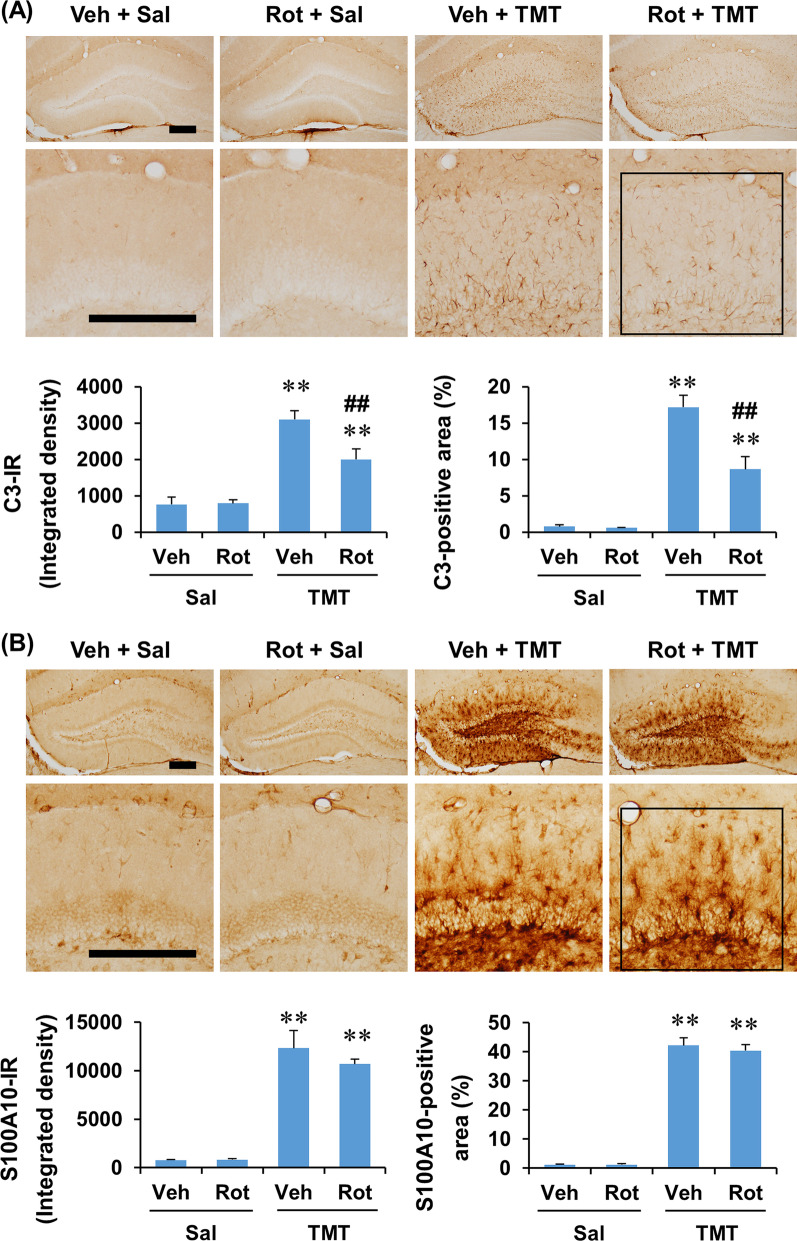
Effect of rottlerin on the expression of C3 and S100A10 in the dentate gyrus of mice 6 days after TMT treatment. **A** Effect of rottlerin on C3 expression. **B** Effect of rottlerin on S100A10 expression. Square boxes in **A** and **B** indicate the region of interest for quantification. Veh, Vehicle. Sal, Saline. Rot, Rottlerin. Each value is the mean ± S.E.M. of 4 (Vehicle + Saline and Rottlerin + Saline) or 5 (Vehicle + TMT and Rottlerin + TMT) mice. ^**^P < 0.01 vs. corresponding Saline; ^##^*P* < 0.01 vs. Vehicle + Saline (two-way ANOVA followed by Fisher’s LSD pairwise comparisons test). Scale bar = 200 µm

Consistently, rottlerin treatment appeared to decrease C3-immunoreactivity in the dentate gyrus 6 days after TMT insult, but seemed not to affect S100A10-immunoreactivity (Fig. 8). In quantitative analysis, two-way ANOVA revealed a significant effect of TMT and rottlerin and a significant TMT × rottlerin interaction on the C3 expression, in terms of integrated density and area. However, two-way ANOVA found a significant effect of only TMT, but not rottlerin, on S100A10 expression (Additional file 1: Table S3, see Additional file 1). Post hoc test indicated that rottlerin significantly decreased C3 expression 6 days after TMT treatment (*P* < 0.01 vs. vehicle + TMT) (Fig. 8). These results suggest that rottlerin attenuates A1 astrocyte polarization rather than astroglial activation induced by TMT.

### PKCδ inhibitor rottlerin attenuated C1q expression induced by TMT in the dentate gyrus of mice

It has been suggested that pro-inflammatory cytokines and C1q released by activated microglia facilitate A1 phenotype polarization of astrocytes [7, 8, 48]. Since rottlerin treatment significantly attenuated A1 astrocyte polarization, but did not significantly affect A2 astrocyte polarization, we next examined the effect of rottlerin on the expression of C1q and representative pro-inflammatory cytokines such as IL-1β and TNFα 6 days after TMT treatment. The basal expression pattern of C1q in the hippocampus is shown in Additional file 2: Fig. S4A (see Additional file 2); C1q-immunoreactivity was moderate around the hippocampal fissure and in the outer molecular layer of the dentate gyrus, and this expression pattern was consistent with previous findings by Stephan et al. [52]. As shown in Additional file 2: Fig. S4B, (see Additional file 2), C1q expression was increased at 2 days, and became more evident at 6 days after TMT treatment in the dentate gyrus. Interestingly, C1q-immunoreactivity, in terms of density, was slightly decreased at 10 days as compared with at 6 days, but C1q expression extended into the stratum lacunosum-moleculare of the hippocampus. In quantitative analysis, ANOVA revealed a significant effect of TMT on C1q expression, in terms of integrated density and area (Additional file 1: Table S5, see Additional file 1). Post hoc test revealed that C1q expression significantly increased at 2 days (area: *P* < 0.05 vs. saline), and became more pronounced at 6 and 10 days after TMT treatment (integrated density and area: *P* < 0.01 vs. saline) (Additional file 2: Fig. S4B, see Additional file 2), indicating that TMT induced a marked increase in C1q expression in the dentate gyrus of mice.

**Fig. 9:**
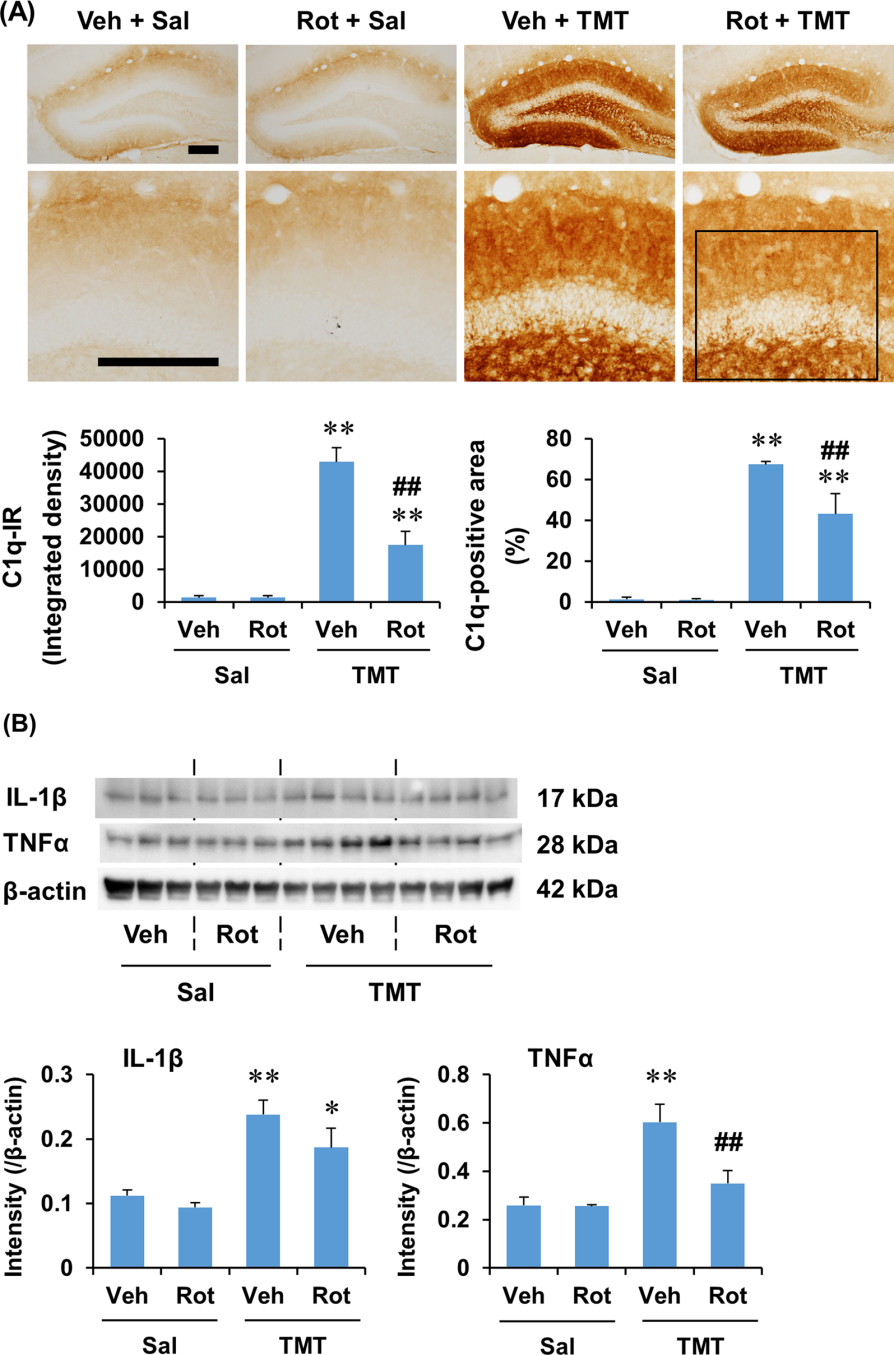
Effect of rottlerin on the expression of C1q, IL-1β, and TNFα in the hippocampus of mice 6 days after TMT treatment. **A** Effect of rottlerin on C1q expression. Each value is the mean ± S.E.M. of 4 (Vehicle + Saline and Rottlerin + Saline) or 5 (Vehicle + TMT and Rottlerin + TMT) mice. **B** Effect of rottlerin on IL-1β and TNFα expression in the hippocampus. Each value is the mean ± S.E.M. of 3 (Vehicle + Saline and Rottlerin + Saline) or 4 (Vehicle + TMT and Rottlerin + TMT) mice. Square boxes in A indicate the region of interest for quantification. Veh, Vehicle. Sal, Saline. Rot, Rottlerin. ^*^P < 0.05, ^**^P < 0.01 vs. corresponding Saline; ^##^*P* < 0.01 vs. Vehicle + Saline (two-way ANOVA followed by Fisher’s LSD pairwise comparisons test). Scale bar = 200 µm

Consistent with the results of PKCδ and Iba-1 expression, rottlerin treatment appeared to decrease TMT-induced C1q expression, and this effect seemed to be more evident in the molecular layer than in the granular cell layer (Fig. 9A). In quantitative analysis, two-way ANOVA indicated the significant effects (integrated density and area) of TMT and rottlerin and a significant rottlerin × TMT interaction (area) on C1q expression (Additional file 1: Table S4, see Additional file 1). Post hoc test indicated that rottlerin significantly attenuated C1q expression at 6 days after TMT treatment (integrated density and area: *P* < 0.01 vs. vehicle + TMT) (Fig. 9A). In Western blot analysis of IL-1β and TNFα expression, the representative pro-inflammatory cytokines, in the hippocampus, two-way ANOVA found a significant effect of TMT on IL-1β and TNFα expression, and indicated a significant effect of rottlerin on TNFα expression, but not on IL-1β expression (Additional file 1: Table S4, see Additional file 1). Consistently, post hoc test revealed that rottlerin significantly attenuated TNFα expression (*P* < 0.01 vs. vehicle + TMT) 6 days after TMT treatment (Fig. 9B), suggesting that the inhibition of C1q and TNFα expression is important for rottlerin-mediated effect on astrocyte polarization after TMT insult in the dentate gyrus of mice.

### PKCδ inhibitor rottlerin attenuated delayed apoptotic cell death induced by TMT in the dentate gyrus of mice

**Fig. 10:**
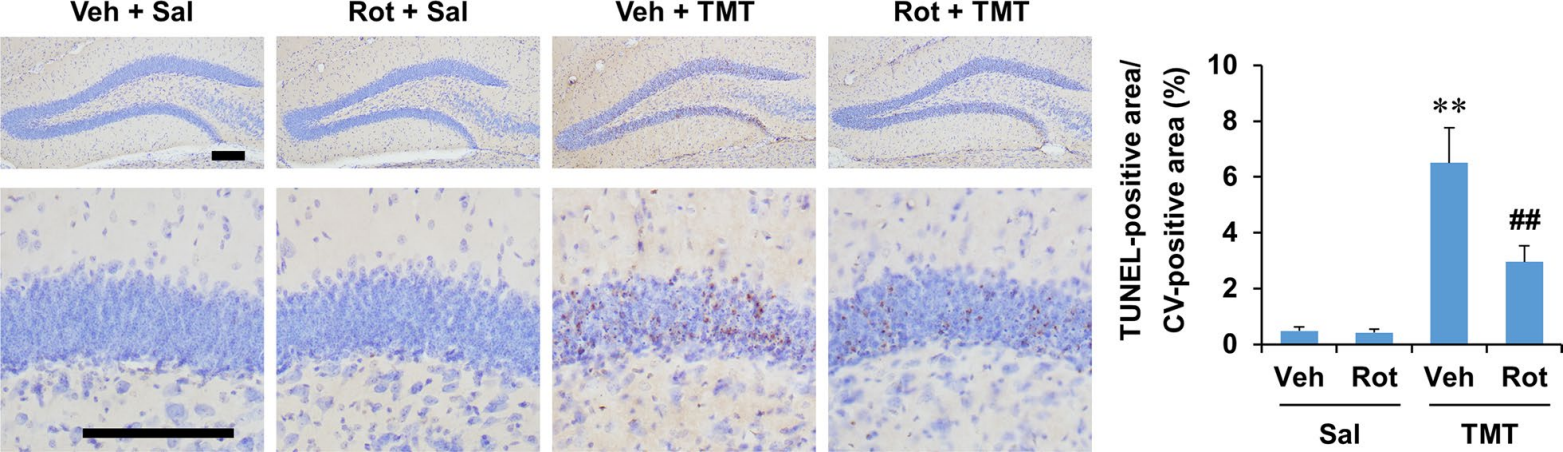
Effect of rottlerin on the delayed apoptotic cell death in the dentate gyrus of mice 6 days after TMT treatment. Apoptotic cell death was examined by TUNEL staining. Veh, Vehicle. Sal, Saline. Rot, Rottlerin. Each value is the mean ± S.E.M. of 4 (Vehicle + Saline and Rottlerin + Saline) or 5 (Vehicle + TMT and Rottlerin + TMT) mice. ^**^P < 0.01 vs. corresponding Saline; ^##^*P* < 0.01 vs. Vehicle + Saline (two-way ANOVA followed by Fisher’s LSD pairwise comparisons test). Scale bar = 200 µm

Our previous study showed that TMT-induced apoptotic cell death peaked at 2 days, but delayed apoptotic cell death continued in the dentate gyrus of mice [38]. Neurotoxic A1 astrocytes have been suggested to induce neuronal apoptosis [48, 53]. Therefore, we next examined whether rottlerin attenuated delayed apoptotic cell death at 6 days after TMT treatment in the dentate gyrus of mice. TUNEL staining showed that rottlerin appeared to decrease the apoptotic cell death in the granular cell layer of the dentate gyrus. In quantitative analysis, two-way ANOVA indicated the significant effects of TMT and rottlerin on TUNEL staining (Additional file 1: Table S4, see Additional file 1). Post hoc test revealed that rottlerin significantly decreased the TUNEL-positive cells at 6 days after TMT treatment (*P* < 0.01 vs. vehicle + TMT) (Fig. 10), suggesting that rottlerin attenuates TMT-induced delayed neuronal apoptosis through inhibition of A1 astrocyte polarization.

## Discussion

In the present study, we characterized the temporal and spatial profiles of astrocyte polarization after TMT insult in the dentate gyrus of mice. We also found that the expression of p-PKCδ, an important mediator of neuroinflammation, was induced in microglial cells concomitant with TMT excitotoxicity. Late- and post-ictal treatment with rottlerin, a PKCδ inhibitor, significantly attenuated microglial activation and decreased the expression of C1q and TNFα, and consequently significantly attenuated A1 astrocyte polarization induced by TMT. Thus, our results suggest that PKCδ is involved in A1 astrocyte polarization through the promotion of microglial activation and subsequent release of pro-inflammatory mediators under excitotoxic conditions.

It has long been demonstrated that astrocytes become reactive in response to various brain injuries and neurodegenerative changes. Reactive astrocytes form a functional barrier in neuropathological conditions, limiting neuroinflammation to the injured area, preventing their spread to adjacent brain tissues, and promoting tissue repair [3–5]. In this regard, the ablation of reactive astrocytes by ganciclovir treatment in GFAP-thymidine kinase transgenic mice has been reported to potentiate neuroinflammation and neurodegeneration in animal models of traumatic brain injury [54, 55] and AD [56]. Accumulating evidence has indicated that reactive astrogliosis in the brain is observed in epileptic patients [57] and in rodent models of epilepsy [58–60]. We have suggested that reactive astrocytes play a protective role by expressing various antioxidant defense components, such as glutathione, glutathione peroxidase, superoxide dismutases, ceruloplasmin, and H-ferritin, in the hippocampus after kainate- or TMT-induced excitotoxicity [61–65]. In contrast, reactive astrogliosis has been shown to facilitate spontaneous recurrent seizures in mice [66, 67], suggesting that the role of reactive astrocytes may be complex under excitotoxic conditions. A time-dependent increase in GFAP protein expression and astroglial activation was also reported after TMT insult in the dentate gyrus of mice [68]. In the present study, we characterized TMT-induced temporal and spatial changes in astroglial activation in the dentate gyrus of mice. Significant astroglial activation was observed at 2 days, and it was more pronounced at 6 and 10 days after TMT treatment.

Recently, it has been reported that reactive astrocytes can be polarized into the pro-inflammatory A1 phenotype or the anti-inflammatory A2 phenotype in various neuropathological conditions [9–11, 13–15]. Although it has been reported that astroglial C3 expression was increased in the hippocampus of epileptic patients [69] or pilocarpine-treated mice [70], these studies did not relate this phenomenon to astrocyte polarization. A recent study done by Wei et al. [16] reported A1 astrocyte polarization and suggested an interaction between A1 astrocytes and microglia after kainate-induced status epilepticus in the hippocampus. In the present study, we characterized the temporal and spatial profiles of astrocyte phenotype polarization after TMT insult in the dentate gyrus of mice. Among A1 phenotype markers, the mRNA expression of C3, CFB, and GGTA1 increased as early as 1 day, peaked at 6 days, and remained increased at 10 and 14 days after TMT treatment. However, MX1 mRNA expression increased specifically at 6 days after TMT injection, supporting the notion that different markers of the same phenotype may have different selectivity and specificity depending on the type and stage of brain pathology [8]. The mRNA expression of A2 phenotype markers, including S100A10, EMP1, and CD109, increased and peaked at 2 days, and gradually decreased to near control levels until 14 days after TMT treatment, indicating that the peak time of mRNA expression of A2 phenotype markers precedes that of A1 phenotype markers.

Immunohistochemical analysis showed that the expression of both C3 and S100A10 peaked at 6 days after TMT treatment in the dentate gyrus of mice. C3 expression remained increased at 10 days, but S100A10 expression significantly decreased at this time point, suggesting that A1 astrocyte polarization lasts longer than A2 astrocyte polarization in this model. Interestingly, C3 mRNA expression started to significantly increase from 1 day after TMT treatment; however, C3 protein expression, as examined by immunohistochemistry, did not significantly increase in astrocytes until 2 days after TMT treatment. Instead, C3 expression appeared to slightly increase in the microvessels 2 days after TMT treatment, which was in line with previous findings showing that C3 expression can be increased by ischemic or inflammatory stimuli in the epithelial cells of brain microvessels [71–73]. Thus, the increase in C3 mRNA expression at early time points (1 and 2 days) could be partly attributed to the increase in non-astroglial C3 mRNA expression induced by TMT. In addition, while C3 protein expression was induced throughout the dentate gyrus, S100A10 expression increased mainly in the hilus, granular layer, and inner molecular layer of the dentate gyrus, reflecting the regional specificity of A1 and A2 astrocyte polarization after TMT insult.

Previous studies have suggested that C3 and S100A10 are specific and exclusive markers of A1 and A2 astrocytes, respectively [48, 49]. Interestingly, in the present study, a significant co-localization of C3 and S100A10 expression (52.3% of C3-positive astrocytes and 42.3% of S100A10-positive astrocytes) was observed 6 days after TMT treatment in the dentate gyrus of mice. Similar findings, in a previous study [74], reported that approximately 32% of the astrocytes in the striatum of aged mice expressed both *C3* and *EMP1* genes in their in situ hybridization study. Recently, it has been shown that A1 and A2 phenotypes can be mutually converted rather than permanently fixed in vitro and in vivo [75–77]. One possibility is that astrocytes expressing both C3 and S100A10 may be in a transition state between the A1 and A2 phenotypes. Another possibility is that polarized astrocytes may predominantly, but not exclusively, express the markers of one phenotype, although this point needs to be further studied. In addition, Liddelow et al. [48] reported that A1 phenotype astrocytes reverted toward a non-reactive phenotype (so-called “A0” astrocytes) upon treatment with transforming growth factor-β or fibroblast growth factor. These findings, together with our present findings, suggest that astrocyte polarization and phenotypic conversion may be more dynamic phenomena than expected.

Activated microglia have been demonstrated to be important for inducing A1 astrocyte polarization through the release of pro-inflammatory factors [7, 8, 11, 48]. Consistently, inhibition of microglial activation using minocycline has been shown to decrease the A1 phenotype astrocytes in a chronic post-surgical pain model [78]. Similarly, ablation of activated microglia by colony stimulating factor 1 receptor (CSF1R) antagonist or *csf1r* gene knockout has been reported to attenuate A1 phenotype polarization induced by kainate excitotoxicity [16] or lipopolysaccharide treatment [48]. In the present study, Iba-1 expression started to significantly increase earlier than C3 or S100A10 expression, suggesting that microglial activation preceded astrocyte polarization after TMT excitotoxicity in the dentate gyrus of mice. This result was in line with a previous finding that the increase in Iba-1 expression preceded that of GFAP or C3 expression after kainate-induced status epilepticus in mice [16]. In contrast, the mRNA expression of GFAP or C3 was shown to increase earlier than the mRNA expression of Iba-1 in the traumatic brain injury model [14], suggesting that the profile of microglial activation and astroglial polarization could be different depending on the type of brain pathology. In addition, although the increase in Iba-1 expression preceded the induction of C3 or S100A10 expression in the present study, the expression levels remained increased until at least 10 days after TMT insult. Thus, the long-term interaction between astrocytes and microglia could be more complex and needs to be explored in future studies. In this context, it has been reported that A1 phenotype astrocytes promote microglial activation by releasing factors that can activate the microglia [8, 16, 79–81], suggesting that microglia and astrocytes form a positive feedback amplifier to enhance neuroinflammation in various neurotoxic conditions.

In the present study, Iba-1 expression was examined to evaluate microglial activation. However, Iba-1 is expressed in monocytes/macrophages as well as in microglia. It has been suggested that peripheral monocytes/macrophages can infiltrate into the brain parenchyma when the blood–brain barrier is disrupted following brain injury and neuroinflammation [82, 83]. Infiltration of monocytes/macrophages into the brain parenchyma has been reported in animal models of stroke [84, 85] and traumatic brain injury [86, 87]. In addition, monocytes/macrophages infiltration has been shown in excitotoxic conditions induced by kainate [88, 89] or pilocarpine [90]. However, it has not yet been reported whether this infiltration occurs after TMT treatment. Therefore, it cannot be excluded that TMT-induced excitotoxicity induces the infiltration of peripheral monocytes/macrophages, which further influences astroglial phenotype polarization. Additional studies are needed for clarification.

PKCδ has been demonstrated to play an important role in neuroinflammation [19, 21, 91, 92]. Consistently, microglial expression of PKCδ and p-PKCδ has been reported in the hippocampus of pilocarpine- or kainate-treated animals [22–25, 92]. Similarly, in the present study, p-PKCδ expression increased mainly in the microglia after TMT excitotoxicity in the dentate gyrus of mice. In addition, we observed that late- and post-ictal treatment with rottlerin, a PKCδ inhibitor, attenuated TMT-induced microglial activation in the dentate gyrus of mice, which was in line with our previous findings that PKCδ inhibition reduced microglial activation and their neurotoxic M1 phenotype polarization in a methamphetamine-induced neurotoxicity model [46, 93].

The present study found that rottlerin treatment decreased the mRNA expression of A1 astrocyte markers, but not A2 astrocyte markers, induced by TMT in the dentate gyrus of mice. Consistently, rottlerin significantly decreased C3-immunoreactivity, but did not significantly affect S100A10-immunoreactivity after TMT insult. Among the pro-inflammatory factors released by activated microglia, C1q, TNFα, and IL-1α have been shown to be critical for the specific induction of astroglial A1 phenotype polarization upon inflammatory stimulus [48]. However, IL-1β, one of the representative inflammatory mediators released by microglia, has shown to be either capable [94] or incapable [48, 95] of inducing A1 astrocyte polarization in primary astrocyte cultures depending on the treatment regimen. Moreover, Shiow et al. [96] showed that the levels of A1 astrocyte transcripts decreased in astrocytes isolated from neonatal mice systemically treated with IL-1β, suggesting that the role of IL-1β is ambiguous in astrocyte phenotype polarization [8]. In the present study, we observed that TMT insult increased the expression of C1q, TNFα, and IL-1β, which was decreased by rottlerin treatment. Therefore, these results suggest that rottlerin-mediated PKCδ inhibition attenuates TMT-induced A1 astrocyte polarization via inhibition of microglial activation and consequent reduction of microglia-derived pro-inflammatory mediators in the dentate gyrus of mice.

It has been suggested that A1 phenotype astrocytes release pro-inflammatory and neurotoxic factors, such as TNFα, IL-1β, nitric oxide, and reactive oxygen species [7, 10], and lose their normal function of promoting neuronal survival [48]. Moreover, several in vitro studies have shown that treatment with A1 astrocyte conditioned medium induces neuronal apoptosis [48, 53, 97]. In the present study, rottlerin treatment significantly attenuated the delayed apoptotic neuronal death induced by TMT in the dentate gyrus of mice. In addition to our previous report that rottlerin attenuates TMT-induced apoptosis by promoting antioxidant defense and neurogenic activities [37, 38], these results suggest that the inhibition of A1 astrocyte polarization may be partly involved in rottlerin-mediated anti-apoptotic effects.

## Conclusions

Our study demonstrated the temporal and spatial profiles of astrocyte polarization after TMT-induced excitotoxicity in the dentate gyrus of mice. In addition, it was found that TMT induced microglial p-PKCδ expression, and that the PKCδ inhibitor rottlerin suppressed the neurotoxic A1 polarization of astrocytes by inhibiting microglial activation and consequent expression of pro-inflammatory factors, including C1q and TNFα. Therefore, these results suggest that PKCδ inhibition might be an important target for suppressing A1 astrocyte polarization and delayed neuronal damage under excitotoxic conditions.

## Supplementary Information

**Additional file 1. **Additional Information and Additional Tables S1–S5.

**Additional file 2. **Additional Figures S1–S5.

## Data Availability

All data generated or analyzed during the current study are available from the corresponding author on reasonable request.

## Abbreviations

AD: Alzheimer’s disease
CFB: Complement factor B
CSF1R: Colony stimulating factor 1 receptor
EMP1: Epithelial membrane protein 1
GGTA1: Glycoprotein galactosyltransferase α1, 3
IL-1α: Interferon-1α
IL-1β: Interleukin-1β
IL-10: Interleukin-10
PD: Parkinson’s disease
PKCδ: Protein kinase Cδ
Real-time RT-PCR: Real-time reverse transcription-polymerase chain
TNFα: Tumor necrosis factor-α
tM: Thresholded Mander’s co-localization coefficient
TMT: Trimethyltin
TUNEL: Terminal deoxynucleotidyl transferase‑mediated dUTP nick end‑labeling
