# Supplementary material for: Effect of rottlerin on astrocyte phenotype polarization after trimethyltin insult in the dentate gyrus of mice
Source: J Neuroinflammation. 2022 Jun 11;19:142. doi: 10.1186/s12974-022-02507-w (PMC9188234; doi:10.1186/s12974-022-02507-w)
Supplement: Supplementary file 1 — Additional file 1. Additional Information and Additional Tables S1–S5. [file 12974_2022_2507_MOESM1_ESM.docx]

**Additional information**

Additional file 1:**Table S1** Primer sequences and annealing temperatures for the real-time RT-PCR analysis.

| Gene | Primer sequences | Annealing temperature (℃) | Gene accession number | References |
| --- | --- | --- | --- | --- |
| C3 | F: 5′-AGCTTCAGGGTCCCAGCTAC-3′  R: 5′-GCTGGAATCTTGATGGAGACGC-3′ | 60.5 | NM_009778.3 | [1] |
| CFB | F: 5′-GAAACCCTGTCACTGTCATTC-3′  R: 5′-CCCCAAACACATACACATCC-3′ | 55.65 | BC005451.1 | [2] |
| GGTA1 | F: 5′-ACCGCCCGGATGTTTTGAC-3′  R: 5′-TGACGTAAAATATGACCCGATGG-3′ | 55.85 | BC006810.1 | [3] |
| MX1 | F: 5′-TCTGAGGAGAGCCAGACGAT-3′  R: 5′-ACTCTGGTCCCCAATGACAG-3′ | 58.4 | BC011113.1 | [4] |
| S100A10 | F: 5′-CCAGGTTTCGACAGACTCTTC-3′  R: 5′-CCGTTCCATGAGCACTCTC-3′ | 57.2 | BC025044.1 |  |
| CD109 | F: 5′-TCTCGTTCTTCCGGCACTAC-3′  R: 5′-GAGTGTGAGCACCCGAAACTT-3′ | 59.15 | DQ832284.1 | [5] |
| EMP1 | F: 5′-GTTGGTGCTACTGGCTGGTC-3′  R: 5′-TACCACCAGTGCAGTTCTTCC-3′ | 59.45 | BC050899.1 | [6] |
| GAPDH | F: 5′-ACCACAGTCCATGCCATCAC-3′  R: 5′-TCCACCACCCTGTTGCTGTA-3′ | 55.45 | GU214026.1 |  |

C3, Complement component 3; CFB, Complement factor B; GGTA1, Glycoprotein galactosyltransferase α1, 3; EMP1, Epithelial membrane protein 1; GAPDH, Glyceraldehyde 3-phosphate dehydrogenase.

Additional file 1:**Table S2** Results of ANOVA for Figs. 1, 2, 4, 5, and 6.

|  | Fig. 1  (C3) | Fig. 1  (CFB) | Fig. 1  (GGTA1) | Fig. 1  (MX1) |
| --- | --- | --- | --- | --- |
| Time points | *F*_5,29_ = 5.564,  *P* = 0.00103^**^ | *F*_5,29_ = 6.759,  *P* = 0.000275^**^ | *F*_5,29_ = 20.287,  *P* = 1.127 × 10^-8**^ | *F*_5,29_ = 8.599,  *P* = 4.345 × 10^-5**^ |
|  | Fig. 1  (S100A10) | Fig. 1  (EMP1) | Fig. 1  (CD109) |  |
| Time points | *F*_5,29_ = 10.527,  *P* = 7.793 × 10^-6**^ | *F*_5,29_ = 53.867,  *P* = 8.543 × 10^-14**^ | *F*_5,29_ = 10.701,  *P* = 6.737 × 10^-6**^ |  |
|  | Fig. 2A  (C3-IR) | Fig. 2A  (C3-positive area) | Fig. 2B  (S100A10-IR) | Fig. 2B  (S100A10-positive area) |
| Time points | *F*_4,15_ = 9.219,  *P* = 0.000575^**^ | *F*_4,15_ = 22.111,  *P* = 3.805 × 10^-6**^ | *F*_4,15_ = 31.472,  *P* = 3.897 × 10^-7**^ | *F*_4,15_ = 269.033,  *P* = 9.135 × 10^-14**^ |
|  | Fig. 4A  (Iba-1-IR) | Fig. 4A  (Iba-1-positive area) | Fig. 4B  (p-PKCδ-IR) | Fig. 4B  (p-PKCδ-positive area) |
| Time points | *F*_4,15_ = 27.931,  *P* = 8.526 × 10^-7**^ | *F*_4,15_ = 25.710,  *P* = 1.458 × 10^-6**^ | *F*_4,15_ = 21.432,  *P* = 4.626 × 10^-6**^ | *F*_4,15_ = 39.524,  *P* = 8.481 × 10^-8**^ |
|  | Fig. 5B  (p-PKCδ-IR) | Fig. 5B  (p-PKCδ-positive area) | Fig. 6A  (Iba-1-IR) | Fig. 6A  (Iba-1-positive area) |
| TMT | *F*_1,14_ = 153.403,  *P* = 6.226 × 10^-9**^ | *F*_1,14_ = 24.846,  *P* = 0.000200^**^ | *F*_1,14_ = 188.275,  *P* = 1.644 × 10^-9**^ | *F*_1,14_ = 223.353,  *P* = 5.343 × 10^-10**^ |
| Rottlerin | *F*_1,14_ = 6.407,  *P* = 0.0240^*^ | *F*_1,14_ = 5.592,  *P* = 0.0330^*^ | *F*_1,14_ = 7.577,  *P* = 0.0156^*^ | *F*_1,14_ = 8.516,  *P* = 0.0112^*^ |
| TMT × Rottlerin | *F*_1,14_ = 2.328,  *P* = 0.149 | *F*_1,14_ = 5.539,  *P* = 0.0337^*^ | *F*_1,14_ = 7.429,  *P* = 0.0164^*^ | *F*_1,14_ = 8.368,  *P* = 0.0118^*^ |

**P* < 0.05, ***P* < 0.01.

Additional file 1: **Table S3** Results of ANOVA for Figs. 7 and 8.

|  | Fig. 7  (C3) | Fig. 7  (CFB) | Fig. 7  (GGTA1) | Fig. 7  (MX1) |
| --- | --- | --- | --- | --- |
| TMT | *F*_1,20_ = 92.071,  *P* = 6.307 × 10^-9**^ | *F*_1,20_ = 63.125,  *P* = 1.296 × 10^-7**^ | *F*_1,20_ = 131.268,  *P* = 3.067 × 10^-10**^ | *F*_1,20_ = 77.570,  *P* = 2.559 × 10^-8**^ |
| Rottlerin | *F*_1,20_ = 5.694,  *P* = 0.0270^*^ | *F*_1,20_ = 7.326,  *P* = 0.0136^*^ | *F*_1,20_ = 1.064,  *P* = 0.315 | *F*_1,20_ = 6.189,  *P* = 0.0218^*^ |
| TMT × Rottlerin | *F*_1,20_ = 5.543,  *P* = 0.0289^*^ | *F*_1,20_ = 6.620,  *P* = 0.0182^*^ | *F*_1,20_ = 1.109,  *P* = 0.305 | *F*_1,20_ = 6.069,  *P* = 0.0229^*^ |
|  | Fig. 7  (S100A10) | Fig. 7  (EMP1) | Fig. 7  (CD109) |  |
| TMT | *F*_1,20_ = 138.852,  *P* = 1.874 × 10^-10**^ | *F*_1,20_ = 32.547,  *P* = 1.391 × 10^-5**^ | *F*_1,20_ = 43.014,  *P* = 2.168 × 10^-6**^ |  |
| Rottlerin | *F*_1,20_ = 1.158,  *P* = 0.295 | *F*_1,20_ = 0.533,  *P* = 0.474 | *F*_1,20_ = 0.282,  *P* = 0.601 |  |
| TMT × Rottlerin | *F*_1,20_ = 0.899,  *P* = 0.354 | *F*_1,20_ = 0.236,  *P* = 0.632 | *F*_1,20_ = 0.256,  *P* = 0.619 |  |
|  | Fig. 8A  (C3-IR) | Fig. 8A  (C3-positive area) | Fig. 8B  (S100A10-IR) | Fig. 8B  (S100A10-positive area) |
| TMT | *F*_1,14_ = 56.876,  *P* = 2.03 × 10^-6**^ | *F*_1,14_ = 82.330,  *P* = 3.071 × 10^-7**^ | *F*_1,14_ = 98.072,  *P* = 1.054 × 10^-7**^ | *F*_1,14_ = 445.593,  *P* = 5.167 × 10^-12**^ |
| Rottlerin | *F*_1,14_ = 5.165,  *P* = 0.0393^*^ | *F*_1,14_ = 10.546,  *P* = 0.00584^**^ | *F*_1,14_ = 0.543,  *P* = 0.473 | *F*_1,14_ = 0.212,  *P* = 0.652 |
| TMT × Rottlerin | *F*_1,14_ = 5.781,  *P* = 0.0306^*^ | *F*_1,14_ = 9.551,  *P* = 0.00798^**^ | *F*_1,14_ = 0.602,  *P* = 0.451 | *F*_1,14_ = 0.236,  *P* = 0.635 |

**P* < 0.05, ***P* < 0.01.

Additional file 1: **Table S4** Results of ANOVA for Figs. 9 and 10.

|  | Fig. 9A  (C1q-IR) | Fig. 9A  (C1q-positive area) | Fig. 9B  (IL-1β) | Fig. 9B  (TNFα) |
| --- | --- | --- | --- | --- |
| TMT | *F*_1,14_ = 71.454,  *P* = 7.171 × 10^-7**^ | *F*_1,14_ = 89.456,  *P* = 1.854 × 10^-7**^ | *F*_1,10_ = 23.627,  *P* = 0.000661^**^ | *F*_1,10_ = 15.318,  *P* = 0.00290^**^ |
| Rottlerin | *F*_1,14_ = 13.967,  *P* = 0.00221^**^ | *F*_1,14_ = 4.614,  *P* = 0.0497^*^ | *F*_1,10_ = 2.368,  *P* = 0.155 | *F*_1,10_ = 5.191,  *P* = 0.0459^*^ |
| TMT × Rottlerin | *F*_1,14_ = 13.990,  *P* = 0.00220^**^ | *F*_1,14_ = 4.320,  *P* = 0.0565 | *F*_1,10_ = 0.515,  *P* = 0.489 | *F*_1,10_ = 4.958,  *P* = 0.0501 |
|  | Fig. 10  (TUNEL) |  |  |  |
| TMT | *F*_1,14_ = 26.448,  *P* = 9.825 × 10^-5**^ |  |  |  |
| Rottlerin | *F*_1,14_ = 4.727,  *P* = 0.0451^*^ |  |  |  |
| TMT × Rottlerin | *F*_1,14_ = 4.379,  *P* = 0.0527 |  |  |  |

**P* < 0.05, ***P* < 0.01.

Additional file 1:**Table S5** Results of ANOVA for Supplementary Figs. S1, S4, and S5.

|  | Supplementary Fig. S1  (GFAP-IR) | Supplementary Fig. S1  (GFAP-positive area) | Supplementary Fig. S4  (C1q-IR) | Supplementary Fig. S4  (C1q-positive area) |
| --- | --- | --- | --- | --- |
| TMT | *F*_4,15_ = 9.089,  *P* = 0.000618^**^ | *F*_4,15_ = 13.749,  *P* = 6.623 × 10^-5**^ | *F*_4,15_ = 36.753,  *P* = 1.385 × 10^-7**^ | *F*_4,15_ = 74.886,  *P* = 9.973 × 10^-10**^ |
|  | Supplementary Fig. S5  (GFAP-IR) | Supplementary Fig. S5  (GFAP-positive area) |  |  |
| TMT | *F*_1,14_ = 16.410,  *P* = 0.00119^**^ | *F*_1,14_ = 29.808,  *P* = 8.409 × 10^-5**^ |  |  |
| Rottlerin | *F*_1,14_ = 0.0134,  *P* = 0.910 | *F*_1,14_ = 0.0334,  *P* = 0.858 |  |  |
| TMT × Rottlerin | *F*_1,14_ = 0.00668,  *P* = 0.936 | *F*_1,14_ = 0.0925,  *P* = 0.766 |  |  |

**P* < 0.05, ***P* < 0.01.

Additional file 1: **References**

1. Clarke LE, Liddelow SA, Chakraborty C, Munch AE, Heiman M, Barres BA. Normal aging induces A1-like astrocyte reactivity. Proc Natl Acad Sci USA. 2018;115(8):E1896-905. <http://doi.org/>10.1073/pnas.1800165115.

2. Sones JL, Merriam AA, Seffens A, Brown-Grant DA, Butler SD, Zhao AM, et al. Angiogenic factor imbalance precedes complement deposition in placentae of the BPH/5 model of preeclampsia. FASEB J. 2018;32(5):2574-86. <http://doi.org/>10.1096/fj.201701008R.

3. Shao A, Xu L, Wu X, Liu S, Lu Y, Fan C. Gal epitope expression and immunological properties in iGb3S deficient mice. Sci Rep. 2018;8(1):15433-7. <http://doi.org/>10.1038/s41598-018-33032-7.

4. Tan YS, Sansanaphongpricha K, Xie Y, Donnelly CR, Luo X, Heath BR, et al. Mitigating SOX2-potentiated Immune Escape of Head and Neck Squamous Cell Carcinoma with a STING-inducing Nanosatellite Vaccine. Clin Cancer Res. 2018;24(17):4242-55. <http://doi.org/>10.1158/1078-0432.CCR-17-2807.

5. Song G, Feng T, Zhao R, Lu Q, Diao Y, Guo Q, et al. CD109 regulates the inflammatory response and is required for the pathogenesis of rheumatoid arthritis. Ann Rheum Dis. 2019;78(12):1632-41. <http://doi.org/>10.1136/annrheumdis-2019-215473.

6. Witkowski MT, Hu Y, Roberts KG, Boer JM, McKenzie MD, Liu GJ, et al. Conserved IKAROS-regulated genes associated with B-progenitor acute lymphoblastic leukemia outcome. J Exp Med. 2017;214(3):773-91. <http://doi.org/>10.1084/jem.20160048.
