# Supplementary material for: Effect of rottlerin on astrocyte phenotype polarization after trimethyltin insult in the dentate gyrus of mice
Source: J Neuroinflammation. 2022 Jun 11;19:142. doi: 10.1186/s12974-022-02507-w (PMC9188234; doi:10.1186/s12974-022-02507-w)

Additional file 2 figure legends

Additional file 2: **Fig. S1** Temporal changes in GFAP expression after TMT treatment in the dentate gyrus of mice. Square box indicates the region of interest for quantification. Sal, Saline. Each value is the mean ± S.E.M. of 4 (Saline, 1 d, 2 d, 6 d, 10 d, and 14 d) mice. ^**^*P* < 0.01 vs. 0 d (one-way ANOVA followed by Fisher’s LSD pairwise comparisons test). Scale bar = 200 µm.


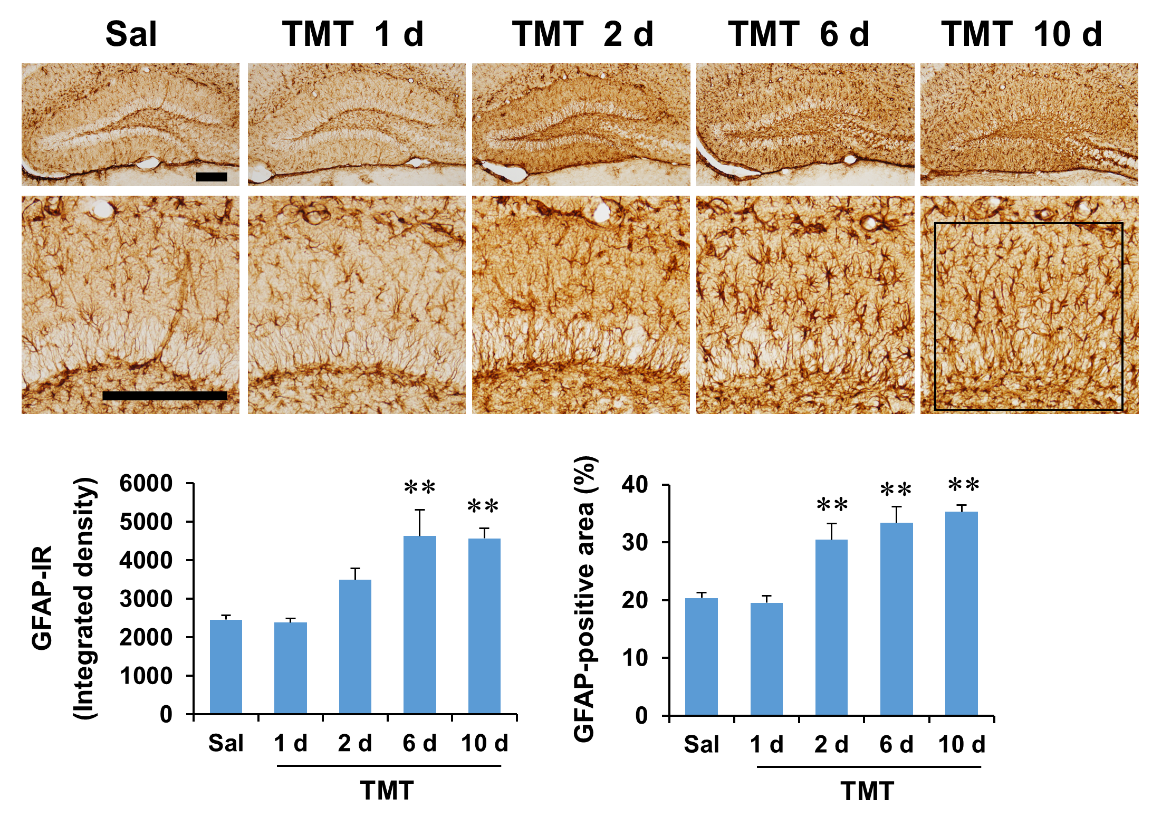


Additional file 2: **Fig. S2** Basal expression pattern of p-PKCδ in the hippocampus of mice. p-PKCδ expression was relatively high in the CA1 subfield (a), moderate in the CA2 and CA3 subfields (b), and negligible in the dentate gyrus (c). Scale bar = 200 µm.

**
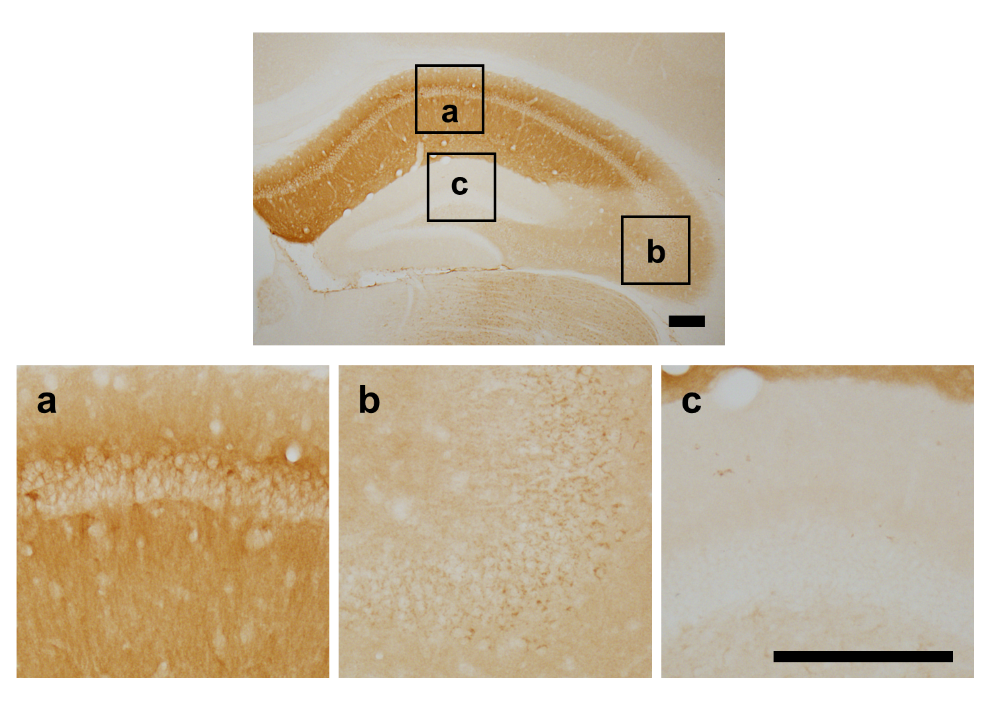
**

Additional file 2: **Fig. S3** Morphology analyses of Iba-1-positive microglia. (A) Cell body size analysis. (B) Cell skeleton analysis. Branches are displayed in orange color. Junction points and branch ends are displayed in purple and blue color, respectively.

**
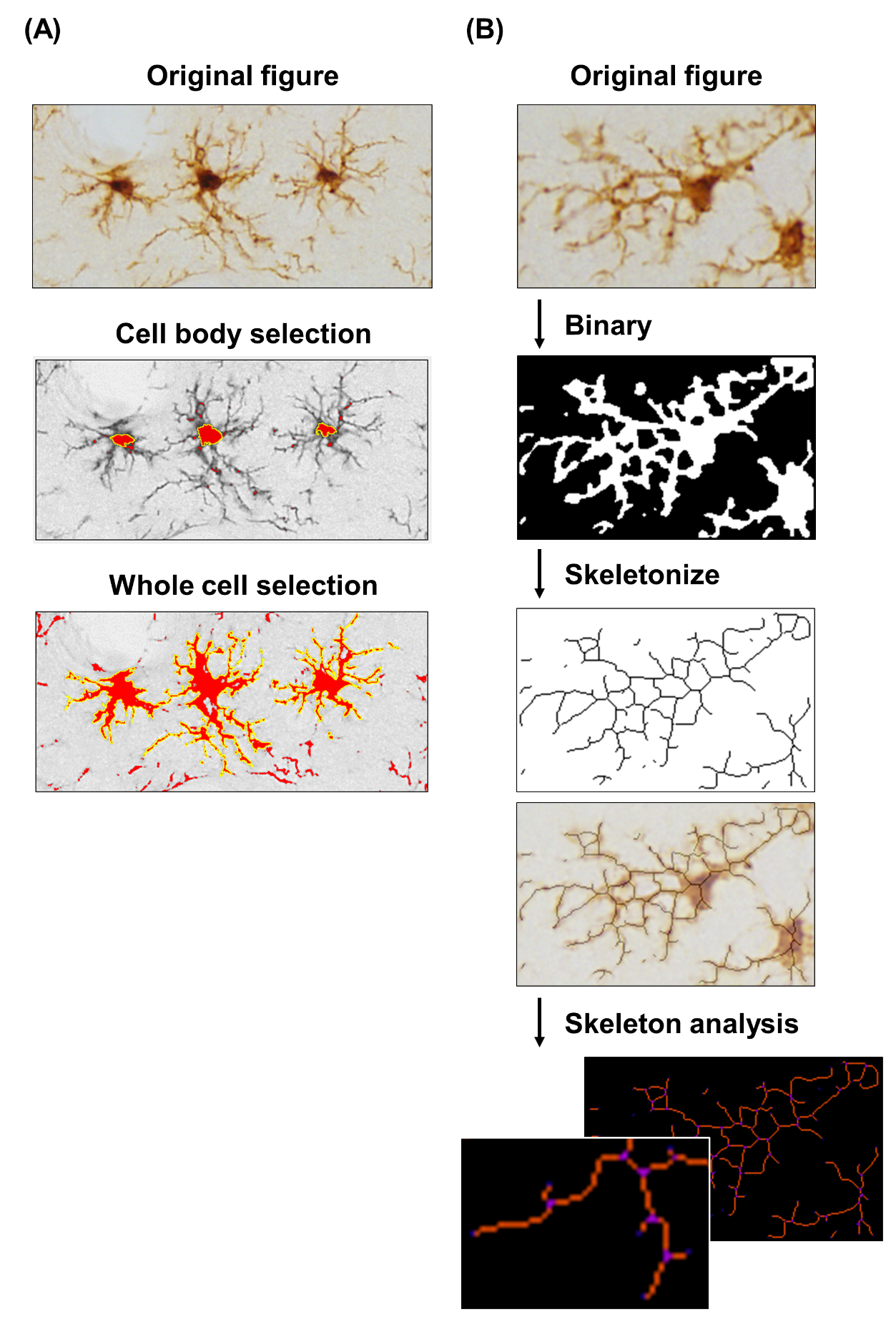
**

Additional file 2: **Fig. S4** Basal expression pattern of C1q in the hippocampus, and temporal changes in C1q expression after TMT treatment in the dentate gyrus of mice. (A) Basal expression of C1q. Moderate C1q-immunoreactivity was observed around the hippocampal fissure and in the outer molecular layer of dentate gyrus (c). The CA1, CA2, and CA3 subfields showed a low immunoreactivity for C1q (a and b). (B) Temporal and spatial changes in C1q expression after TMT treatment in the dentate gyrus of mice. Square box indicates the region of interest for quantification. Sal, Saline. Each value is the mean ± S.E.M. of 4 (Saline, 1 d, 2 d, 6 d, 10 d, and 14 d) mice. ^**^*P* < 0.01 vs. Saline (one-way ANOVA followed by Fisher’s LSD pairwise comparisons test). Scale bar = 200 µm.


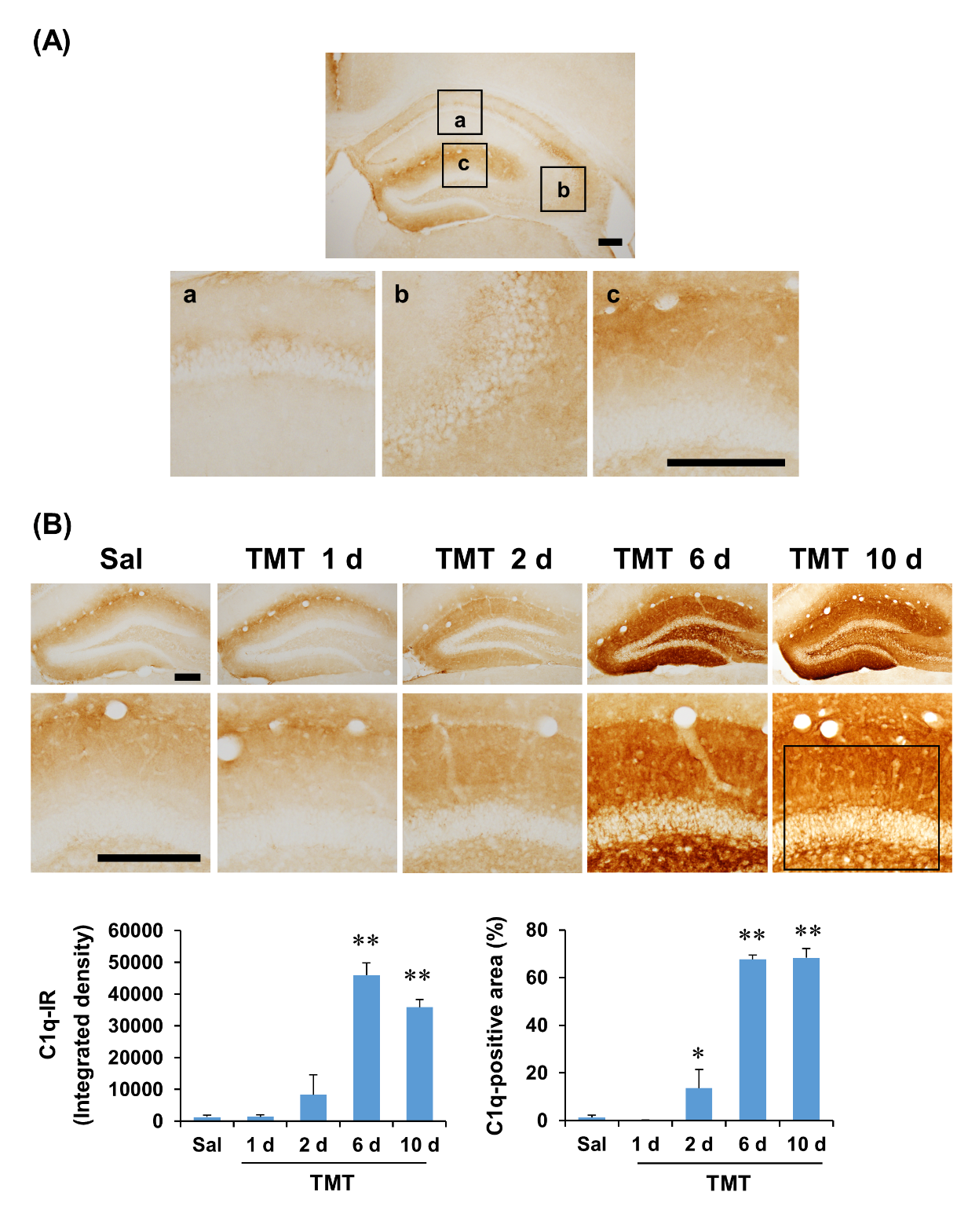


Additional file 2: **Fig. S5** Effect of rottlerin on GFAP expression in the dentate gyrus of mice 6 days after TMT treatment. Square box indicates the region of interest for quantification. Veh, Vehicle. Sal, Saline. Rot, Rottlerin. Each value is the mean ± S.E.M. of 4 (Vehicle + Saline and Rottlerin + Saline) or 5 (Vehicle + TMT and Rottlerin + TMT) mice. ^*^*P* < 0.05, ^**^*P* < 0.01 vs. corresponding Saline (two-way ANOVA followed by Fisher’s LSD pairwise comparisons test). Scale bar = 200 µm.


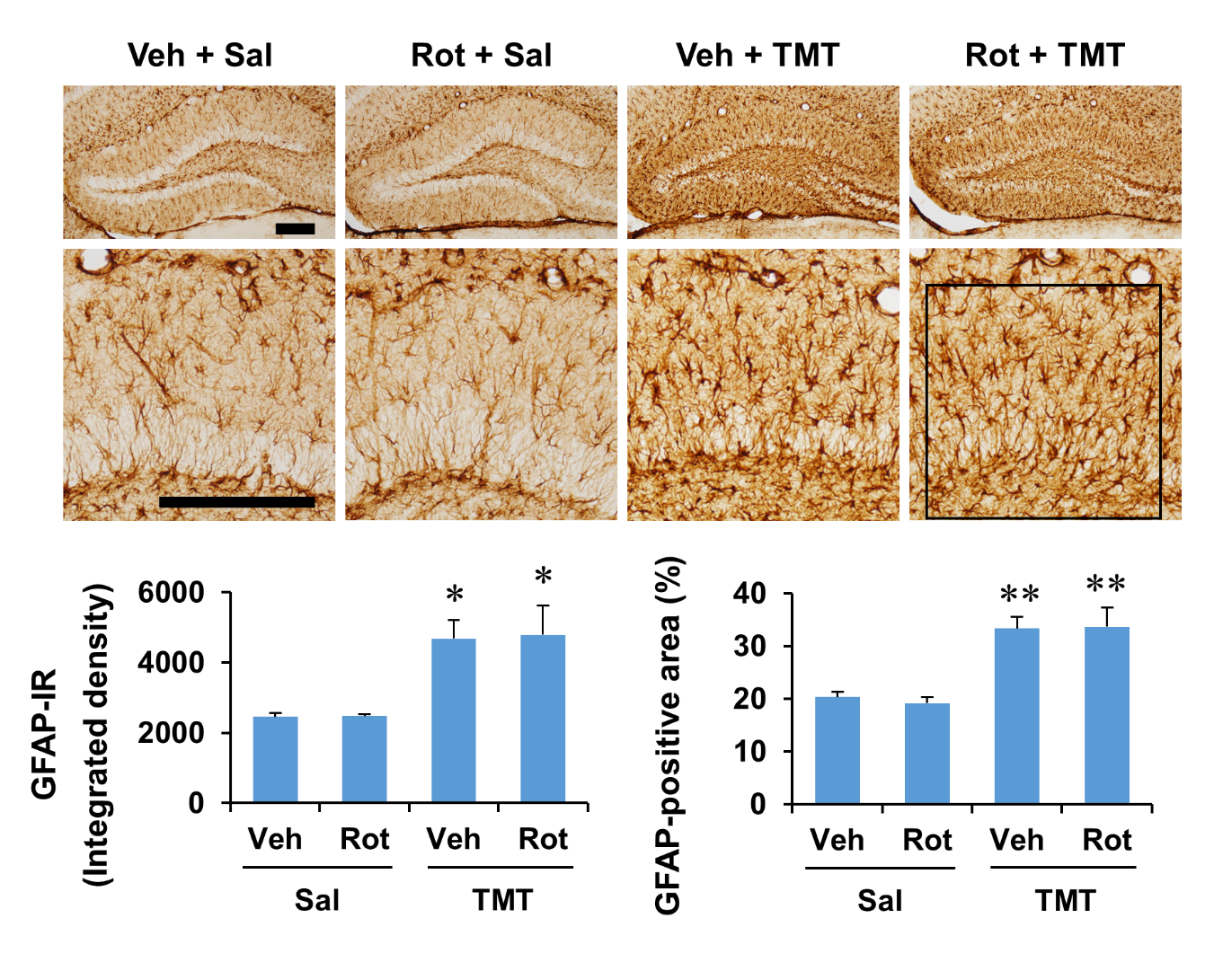

Supplement: Supplementary file 2 — Additional file 2. Additional Figures S1–S5. [file 12974_2022_2507_MOESM2_ESM.docx]
